# BaTiO_3_ Nanoparticle-Induced Interfacial Electric Field Optimization in Chloride Solid Electrolytes for 4.8 V All-Solid-State Lithium Batteries

**DOI:** 10.1007/s40820-025-01901-2

**Published:** 2025-09-01

**Authors:** Qingmei Xiao, Shiming Huang, Donghao Liang, Cheng Liu, Ruonan Zhang, Wenjin Li, Guangliang Gary Liu

**Affiliations:** Guangdong Provincial Key Laboratory of New Energy Materials Service Safety, College of Materials Science and Engineering, Shenzhen University, Shenzhen, 518060 People’s Republic of China

**Keywords:** All-solid-state batteries, Chloride electrolyte, Ferroelectric BaTiO_3_, High-voltage stability, Surface modification

## Abstract

Chloride-based solid electrolytes are considered promising candidates for next-generation high-energy–density all-solid-state batteries (ASSBs). However, their relatively low oxidative decomposition threshold (~ 4.2 V vs. Li^+^/Li) constrains their use in ultrahigh-voltage systems (e.g., 4.8 V). In this work, ferroelectric BaTiO_3_ (BTO) nanoparticles with optimized thickness of ~ 50–100 nm were successfully coated onto Li_2.5_Y_0.5_Zr_0.5_Cl_6_ (LYZC@5BTO) electrolytes using a time-efficient ball-milling process. The nanoparticle-induced interfacial ionic conduction enhancement mechanism contributed to the preservation of LYZC’s high ionic conductivity, which remained at 1.06 mS cm^−1^ for LYZC@5BTO. Furthermore, this surface electric field engineering strategy effectively mitigates the voltage-induced self-decomposition of chloride-based solid electrolytes, suppresses parasitic interfacial reactions with single-crystal NCM811 (SCNCM811), and inhibits the irreversible phase transition of SCNCM811. Consequently, the cycling stability of LYZC under high-voltage conditions (4.8 V vs. Li⁺/Li) is significantly improved. Specifically, ASSB cells employing LYZC@5BTO exhibited a superior discharge capacity of 95.4 mAh g^−1^ over 200 cycles at 1 C, way outperforming cell using pristine LYZC that only shows a capacity of 55.4 mAh g^−1^. Furthermore, time-of-flight secondary ion mass spectrometry and X-ray photoelectron spectroscopy analysis revealed that Metal-O-Cl by-products from cumulative interfacial side reactions accounted for 6% of the surface species initially, rising to 26% after 200 cycles in pristine LYZC. In contrast, LYZC@5BTO limited this increase to only 14%, confirming the effectiveness of BTO in stabilizing the interfacial chemistry. This electric field modulation strategy offers a promising route toward the commercialization of high-voltage solid-state electrolytes and energy-dense ASSBs.

**Supplementary Information:**

The online version contains supplementary material available at 10.1007/s40820-025-01901-2.

## Highlights

- Time efficient ball milling achieves uniform BaTiO_3_ ( coating without sacrificing ionic conductivity (1.06 mS cm^−1^).
- Ferroelectric BTO coating suppresses Li_2.5_Y_0.5_Zr_0.5_Cl_6_ (LYZC decomposition at 4.8 V via electric field modulation, enabling 76% capacity retention after 150 cycles.
- BTO effectively minimizes the formation of interfacial ZrCl_3_O /YCl_2_O byproducts and mitigates the irreversible phase transition of single crystal NCM811 (SCNCM811), thereby improving the compatibility between LYZC and SCNCM811.

**Supplementary Information:**

The online version contains supplementary material available at 10.1007/s40820-025-01901-2.

## Graphical abstract

**Figure:**
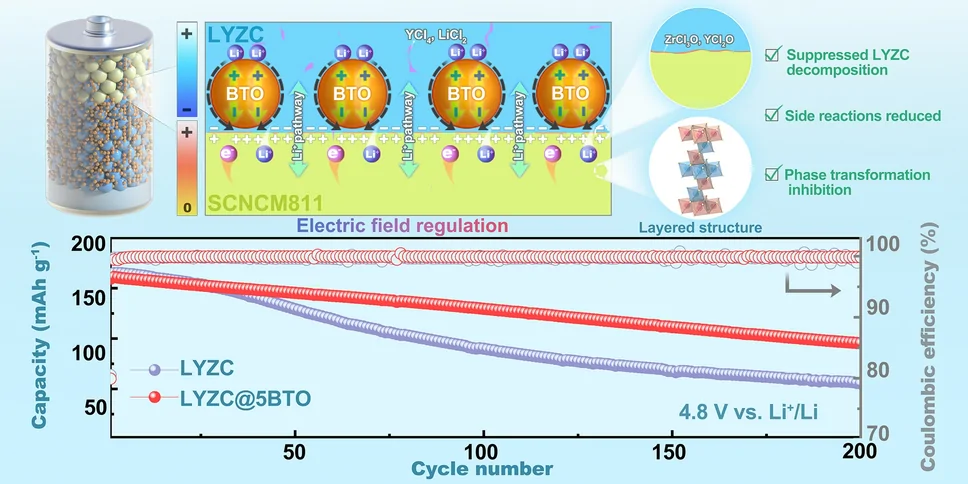

## Introduction

All-solid-state batteries (ASSBs) offer enhanced safety over conventional lithium-ion systems by using non-flammable solid electrolytes (SEs) [1–3]. These SEs resist lithium dendrite propagation and have high oxidative stability, allowing for the use of high-capacity lithium metal anodes and ultrahigh-voltage cathode materials [4–6]. ASSBs can achieve energy densities exceeding 500 Wh kg^−1^, making them ideal for applications like spacecraft propulsion, robotics, and next-gen electric vehicles [7–9]. However, developing commercially viable ASSBs require solid-state electrolytes with lithium-ion conductivities above 1 mS cm^−1^ at 25 °C and electrochemical stability over 4.5 V versus Li^+^/Li [10].

- Time-efficient ball-milling achieves uniform BaTiO_3_ (BTO) coating without sacrificing ionic conductivity (1.06 mS cm^−1^).
- Ferroelectric BTO coating suppresses Li_2.5_Y_0.5_Zr_0.5_Cl_6_ (LYZC) decomposition at 4.8 V via electric field modulation, enabling 76% capacity retention after 150 cycles.
- BTO effectively minimizes the formation of interfacial ZrCl_3_O/YCl_2_O by-products and mitigates the irreversible phase transition of single-crystal NCM811 (SCNCM811), thereby improving the compatibility between LYZC and SCNCM811.

Inorganic solid electrolytes offer excellent ionic transport and broad electrochemical windows, making them suitable for advanced energy storage systems [11]. Key inorganic SE families include oxide-, chloride-, and fluoride-based systems, each with distinct anion-dependent electrochemical behaviors [12–14]. Fluoride electrolytes exhibit high oxidation resistance but limited ionic conductivity (< 10^–7^ S cm^−1^ at 25 °C) [15]. Sulfide systems provide high ionic mobility but decompose above 2.3 V, restricting their application in high-voltage scenarios [16]. Chloride solid electrolytes (CSEs) balance voltage tolerance (~ 4.2 V) and ionic conduction (> 1 mS cm^−1^) [17, 18], demonstrated by compositions like Li_3_InCl_6_ (LIC) and Li_2.5_Y_0.5_Zr_0.5_Cl_6_ (LYZC), which are compatible with commercial 4.3 V-class layered oxide cathodes (e.g., LiCoO_2_ (LCO), LiNi_0.8_Co_0.1_Mn_0.1_O_2_ (NCM811)) [19–21]. However, under extreme high-voltage conditions (> 4.5 V) with high-nickel or Li-rich cathodes, CSEs are susceptible to accelerated electrochemical decomposition and interfacial degradation mechanisms [22, 23]. It is reported by Nazar et al. that LYZC reacts with deeply delithiated Li_*x*_Ni_0.85_Co_0.1_Mn_0.05_O_2_ cathodes, forming insulating YOCl phases during cycling, which exacerbates cell capacity decay [24].

Recent advancements in the engineering of CSEs have focused on three approaches to address high-voltage instability. The first approach, known as the high-entropy cation substitution strategy, is exemplified by Huang et al.’s multi-cation Li_2.75_Y_0.16_Er_0.16_Yb_0.16_In_0.25_Zr_0.25_Cl_6_ design, which utilizes lattice distortion effects to suppress Cl^−^ oxidation kinetics. This method extends the electrochemical stability window to 4.6 V while maintaining an ionic conductivity of 1.17 mS cm^−1^ [25]. The second approach is the complementary fluorine doping technique, demonstrated by Sun et al. in their Li_3_InCl_4.8_F_1.2_ compound [26]. This technique promotes the in situ formation of fluorinated cathode–electrolyte interphases (CEIs). These CEIs passively develop reactive surfaces, enabling them to withstand oxidative potentials up to 4.8 V [26]. Finally, the heterogeneous phase engineering approach, pioneered by Duan et al., utilizes nanocomposite architectures, such as AlOCl-nano LiCl hybrids, to enhance interfacial ion transport and achieve oxidative stability. When combined with Li-rich cathodes, specifically Li_1.17_Mn_0.55_Ni_0.24_Co_0.05_O_2_, 4.8 V versus Li^+^/Li, these architectures show long-term cycling stability (> 1000 cycles) [27].

Although these strategies improve the compatibility of CSEs with high-voltage cathodes exceeding 4.5 V, significant challenges remain in balancing high raw material costs, scalable synthesis processes, and interfacial dynamic stability over extended cycling periods. Therefore, further research into structural engineering strategies using simplified methodologies is necessary.

Ferroelectric materials such as barium titanate (BaTiO_3_, BTO) exhibit distinctive behaviors in an electric field environment. When an electric field is applied, ions within BTO (e.g., Ba^2+^ and Ti^4+^) are induced to deviate from their equilibrium positions, thereby generating instantaneous dipole moments. These dipoles align with the direction of the applied electric field and produce a counteracting reverse electric field. This intrinsic property enables BTO to effectively modulate the electric field distribution at the interfaces between organic/inorganic SEs and layered cathode materials, thus influencing surface Li^+^ conduction behavior and interfacial stability [28, 29]. Notably, BTO can reduce the interfacial impedance between organic and inorganic SEs and layered cathodes by suppressing the space charge layer (SCL) effect. For instance, BTO nanoparticles construct a fast, continuous Li^+^ conduction pathway on the surface of LCO cathodes, improving Li^+^ migration kinetics when paired with Li_6_PS_5_Cl [30]. Additionally, BTO mitigates the concentration of interfacial electric fields under high-voltage conditions, enhancing oxidation resistance. Wu et al*.* incorporated BTO into polymer electrolytes to enhance their compatibility with high-voltage LCO [31]. Surface electric field engineering/regulating offers potential for boosting CSEs oxidative stability but requires more investigation.

This study introduced low-cost BTO nanoparticles on the surface of LYZC solid electrolytes using a straightforward high-energy ball-milling process. Detailed characterization of ion transport kinetics revealed that the addition of inert BTO could modulate lithium-ion transport on the electrolyte surface without affecting the initial ion transport capacity. At an ultrahigh voltage of 4.8 V (vs. Li^+^/Li), the ASSBs employing single-crystal NCM811 (SCNCM811) and LYZC with 5 wt.% BTO coating (LYZC@5BTO) showed a high initial specific capacity and superior rate performance. Kelvin probe force microscopy (KPFM) and cyclic voltammetry (CV) indicated that BTO effectively regulated the electric field distribution on the LYZC surface, suppressing the self-decomposition of LYZC at 4.8 V. Additionally, time-of-flight secondary ion mass spectrometry (ToF–SIMS) and electrochemical impedance spectroscopy (EIS) results demonstrated that LYZC@5BTO significantly reduced surface side reactions compared to bare LYZC. The X-ray diffraction (XRD) refinement and high-resolution transmission electron microscopy (HRTEM) analyses of the cathode/electrolyte composite layer after cycling further confirm the enhanced efficacy of the BTO-modified layer in suppressing irreversible phase transitions at the SCNCM811 surface, as well as in preserving the structural integrity of both SCNCM811 and LYZC under high-voltage conditions. This combination of chemical modification and surface electric field regulation enabled the ASSBs to achieve excellent electrochemical performance and suggested a novel strategy for designing solid electrolytes with high oxidation stability and a stable cathode-electrolyte interphase by modulating the surface electric field.

## Experimental Section

### Preparation of Electrolytes

**Fig. 1:**
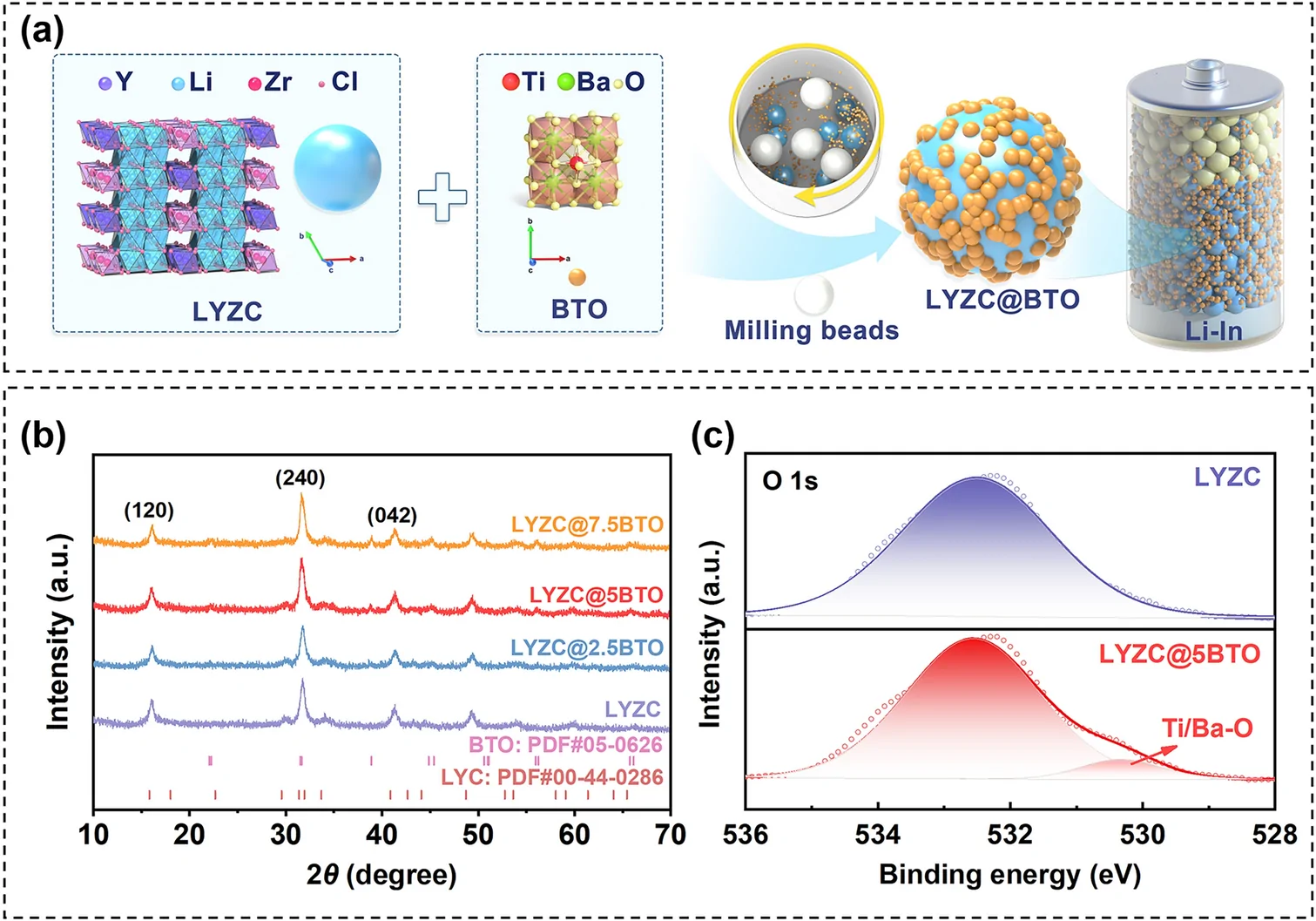
**a** Schematic illustration of compositions and preparation process of LYZC@xBTO. **b** XRD patterns of LYZC@*x*BTO (*x* = 0, 2.5, 5, and 7.5). **c** XPS spectra of O 1*s* for LYZC and LYZC@5BTO electrolytes

Li_2.5_Y_0.5_Zr_0.5_Cl_6_ (LYZC) was obtained from Shenzhen Yanpin Technology Co., Ltd. BaTiO_3_ nanoparticles (supplied by Buwei Co. Ltd., with a purity of 99.9%) were selected as the ferroelectric coating material. A high-energy ball mill (Changsha Miqi Instrument and Equipment Co., Ltd.), as shown in Fig. 1a, was utilized for the coating process, with BTO to LYZC mass ratios of 0, 2.5, 5, 7.5, 10, and 20 wt.%. The ball mill operated at 550 r min^−1^ for 10 min for each test, with ZrO_2_ grinding media (diameter: 5 mm, purity > 99.9%) to LYZC/BTO mixture in a mass ratio of 30:1. The modified electrolytes were designated as LYZC, LYZC@2.5BTO, LYZC@5BTO, LYZC@7.5BTO, LYZC@10BTO, and LYZC@20BTO, respectively.

### Materials Characterization

Phase identification and crystal structure analysis were conducted with a Rigaku SmartLab X-ray diffractometer (XRD), using Cu-Kα radiation from 10° to 70° at 3° min^−1^ scanning rate, under 40 kV and 30 mA operating conditions. XRD data were refined with GSAS-II software via the Rietveld method. Morphological and microstructural characteristics of the samples were investigated using a field-emission scanning electron microscope (SEM, Hitachi SU-70) equipped with an energy-dispersive X-ray spectroscopy (EDS) system. Atomic-level characterization was performed via transmission electron microscopy (TEM, JEOL F200) operated at 200 kV. Electrolyte surface potential was measured by Kelvin Probe Force Microscopy (KPFM, Bruker Dimension® Icon). Elemental composition and chemical states of the samples were characterized by X-ray photoelectron spectroscopy (XPS) measurements conducted on a Thermo ESCALAB 250 system with a monochromatic Al-Kα X-ray source (1486.6 eV).

Time-of-flight secondary ion mass spectrometry (ToF–SIMS) analyses were performed using a TOF–SIMS 5–100 system (IONTOF). Sample preparation was conducted in an Ar-filled glovebox. Composite cathodes were attached to the sample holder using insulating adhesive tape. Sample transfer to the instrument was achieved under an inert atmosphere using a Leica EM VCT500 transfer system (Leica Microsystems). All measurements were performed in negative ion mode with Bi^3+^ primary ions (25 keV). Low-energy electrons were employed for charge compensation during analysis. A cycle time of 60 µs was maintained in all cases. Surface analysis was conducted in spectrometry mode (bunched mode) to ensure high signal intensities and high mass resolution.

### Ion Conductivity and Electrochemical Measurements

The ionic conductivity of the solid electrolyte was evaluated using electrochemical impedance spectroscopy (EIS). Solid electrolyte powders were compressed into pellets with a diameter of 10 mm. These pellets were placed between two stainless steel blocking electrodes and sealed in a model cell within an argon-filled glove box. EIS measurements were conducted using the Princeton Electrochemical Workstation (PARSTAT3000A-DX), covering a frequency range from 1 MHz to 10 mHz and a voltage amplitude of 10 mV at various temperatures (0, 10, 20, 30, 40, 50, 60, 70, 80, 90, 100, and 110 °C). The ionic conductivity (*σ*) and activation energy (*Eₐ*) were determined using Eqs. (1) and (2) [32, 33]:

1 $$\sigma =\frac{d}{{R}_{\mathrm{total}}S}$$

where *d*, *S*, and *R*_total_ represent the sample thickness, effective area of the electrode (0.0785 cm^2^), and total impedance of the sample, respectively.

2 $${E}_{a}(\mathrm{eV})={K}_{B}T\mathrm{ln}(\frac{A}{\sigma })$$

where *A* denotes the pre-exponential factor, *T* indicates temperature in Kelvin, and *K*_*B*_ is the Boltzmann constant (8.617 × 10^–5^ eV K^−1^).

For cyclic voltammetry (CV) tests, LYZC and LYZC@5BTO powders were manually mixed with carbon nanofibers at a mass ratio of 9: 1 using a mortar and pestle. A quantity of 100 mg of the solid electrolyte was pressed into a pellet within a PTFE cylinder with an inner diameter of 10 mm, utilizing two stainless steel rods under a pressure of 150 MPa. Subsequently, 10 mg of the LYZC@*x*BTO (*x* = 0 or 5)/carbon composite was deposited onto one side of the pellet under a compaction pressure of 300 MPa. A 100-mg LiIn alloy foil (In:Li = 98:2) was then placed on the opposite side, serving as both the counter and reference electrodes.

For the fabrication of ASSBs, the cathode composite was prepared by manually grinding 70 mg of SCNCM811 cathode material with 30 mg of LYZC@*x*BTO (*x* = 0, 2.5, 5, 7.5) solid electrolyte in a mortar using a pestle. Subsequently, 100 mg of LYZC@*x*BTO solid electrolyte was placed into a 10-mm-diameter mold and cold-pressed at 150 MPa to form a dense solid electrolyte layer. A uniform layer of 6–8 mg of the cathode composite was then spread onto the surface of the solid electrolyte layer and cold-pressed at 300 MPa for one minute to ensure intimate contact. The LiIn alloy anode was subsequently cold-pressed onto the opposite side of the electrolyte layer at 150 MPa for 3 min. Aluminum and copper foils were used as current collectors for the positive and negative electrodes, respectively. All components were assembled and sealed within a model cell inside an Ar-filled glove box to maintain an inert atmosphere. The mass loading of the cathode material was approximately 5.8 mg cm^−2^. Galvanostatic charge–discharge measurements were conducted on a LAND battery test system within the voltage ranges of 2.8–4.3, 2.8–4.8, and 2.8–5 V vs. Li^+^/Li. The charge–discharge rates varied between 0.1, 0.2, 0.5, 1, and 2 C, where 1 C corresponds to a current density of 200 mA g^−1^.

## Results and Discussion

### Structural and Morphological Characterization of LYZC@BTO

LYZC was chosen as the solid electrolyte due to its low-cost constituents, Zr (~ 22 USD kg^−1^) and Y (~ 43 USD kg^−1^), aligning with Panasonic’s scalable chloride–electrolyte strategy [34]. BTO nanoparticle (111 USD kg^−1^) was used as an interfacial modifier for its cost-efficient ferroelectric properties to stabilize cathode–electrolyte interfaces, as previously demonstrated in polymer battery systems [35]. Figure 1a not only depicts the schematic diagram of the ball-milling process but also presents the crystal structures of both LYZC and BTO materials. Specifically, LYZC crystallizes in the trigonal P-3m1 space group, where Li^+^ ions conduct along the c-axis while Y^3+^ and Zr^4+^ are coordinated by Cl^−^. In contrast, BaTiO_3_ exhibits a perovskite structure with tetragonal symmetry (P4mm) at room temperature, comprising Ba^2+^, Ti^4+^, and O^2−^ ions.

Structural changes in LYZC before and after different amount BTO incorporation (LYZC@*x*BTO, *x* = 0, 2.5, 5, 7.5) were investigated via XRD. All XRD patterns are indexed to the trigonal P-3m1 space group (JCPDS card No. 44-0286) [36], showing no impurity, as shown on Fig. 1b. At the same time, characteristic BTO peaks at 2*θ* = 22.3°, 38.9°, and 56.1° became increasingly pronounced as the BTO loading increased, which confirms successful integration of the BTO modifier [37]. Rietveld refinement of the experimental data (Fig. S1) yielded weighted profile residuals (wR) < 10% for all samples, showing good agreement between observed and calculated patterns [38]. As summarized in Table S1, lattice parameter c/a ratios exhibited no significant variation across all samples, indicating that the BTO coating did not change the crystalline structure of the LYZC electrolyte.

XPS was used to analyze the surface chemistry of LYZC and LYZC@5BTO, with the latter chosen for its optimal electrochemical performance. High-resolution XPS spectra for O 1*s*, Ti 2*p*, and Ba 3*d* orbitals are shown in Figs. 1c and S2. Figure 1c illustrates a peak at 530.2 eV in the O 1*s* spectrum of LYZC@5BTO, corresponding to Ti/Ba–O bonds [39], confirming successful BTO integration into the LYZC surface. No notable changes were detected in the Y 3*d*, Zr 3*d*, or Cl 2*p* spectra between pristine LYZC and LYZC@5BTO samples (Fig. S3), suggesting that BTO modification does not affect the chemical environment of the LYZC matrix. These findings support XRD results, indicating that BTO inclusion does not cause structural disruption or phase impurities.

**Fig. 2:**
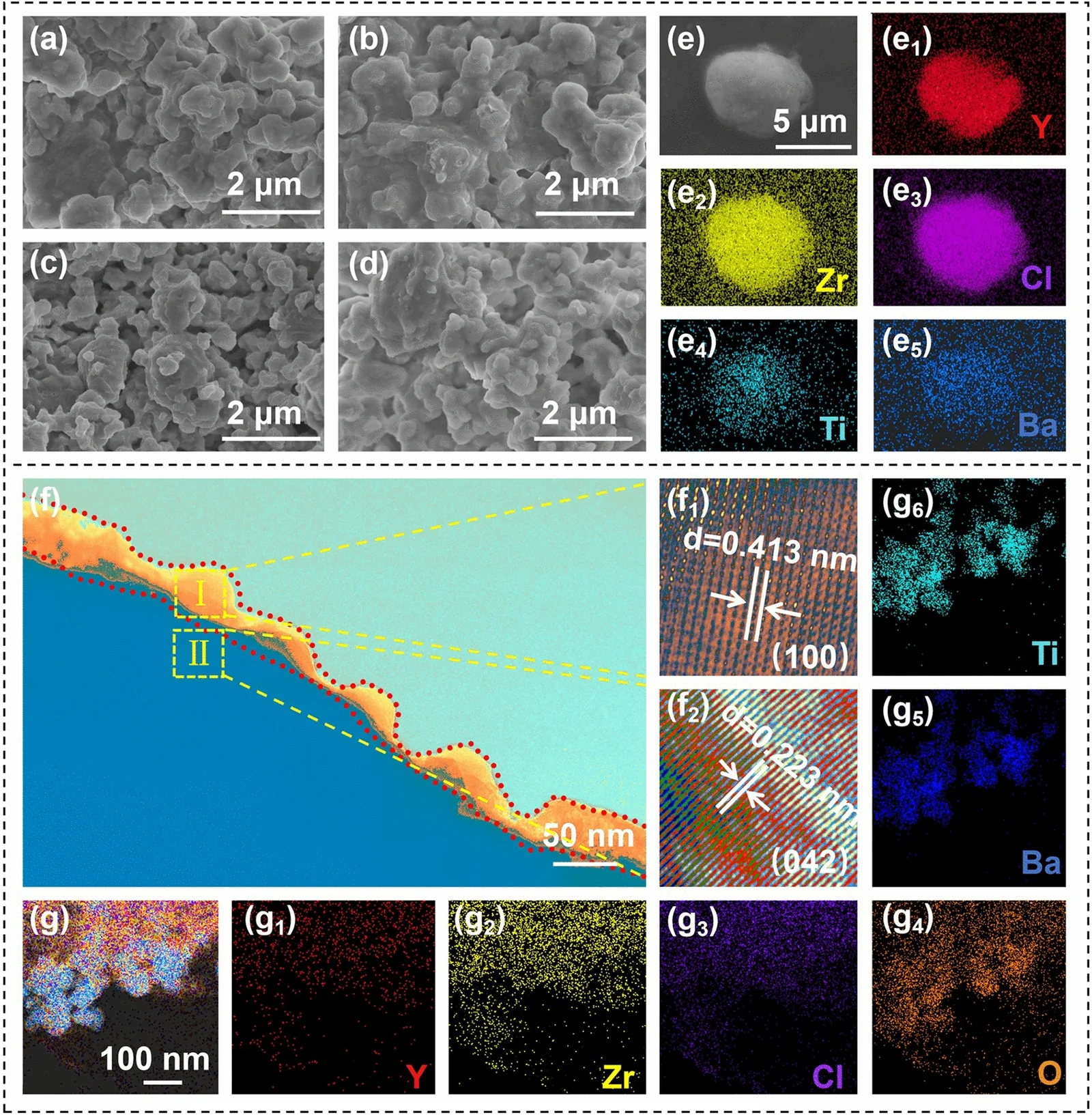
SEM images of the LYZC@*x*BTO **a** *x* = 0, **b** *x* = 2.5, **c** *x* = 5, and **d** *x* = 7.5. **e** SEM image of LYZC@5BTO with corresponding EDS elemental mapping of **(e**_**1**_**-e**_**5**_**)** Y, Zr, Cl, Ti, and Ba, respectively. **f** TEM images of LYZC@5BTO with **(f**_**1**_**, f**_**2**_**)** high-resolution lattice plane images. **g** TEM images of LYZC@5BTO with corresponding EDS elemental mappings of **(g**_**1**_**-g**_**6**_**)** Y, Zr, Cl, O, Ba, and Ti, respectively

The morphological and microstructural changes of LYZC before and after BTO modification were analyzed using SEM and high-resolution TEM (HRTEM). SEM images of commercial BTO nanoparticles (Fig. S4) show nearly spherical particles with an average diameter of ~ 50 nm. SEM images of LYZC@*x*BTO and ball-milled pristine LYZC (Fig. 2a-d) show similar particle sizes (2–3 μm), indicating that BTO modification does not cause significant agglomeration or size reduction. EDS maps of a representative LYZC@5BTO particle (Fig. 2e1–e5) reveal homogeneous distribution of Ti, Ba, and O across the surface and uniform distribution of Y, Zr, and Cl throughout, confirming successful integration of BTO onto the LYZC surface.

HRTEM analysis (Figs. 2f and S5, S6) further corroborates the core–shell structure, revealing a ~ 50–100-nm-thick BTO coating layer that encapsulates the LYZC particle. Lattice fringes with interplanar spacings of 0.413 and 0.223 nm (Fig. 2f1, f2) correspond to the (100) plane of BTO and the (042) plane of LYZC, respectively [40, 41]. EDS mapping of the interfacial region (Fig. 2g1–g6) confirms the colocalization of Ti, Ba, and O within the coating layer, with minimal diffusion into the LYZC matrix. Overlay analysis of Ba, Ti, O, and Cl elemental maps (Fig. 2g) further indicates slight thickness variations in the BTO coating, with an average ranging from 50 to 100 nm.

### Lithium-Ion Transport Properties of LYZC@BTO

**Fig. 3:**
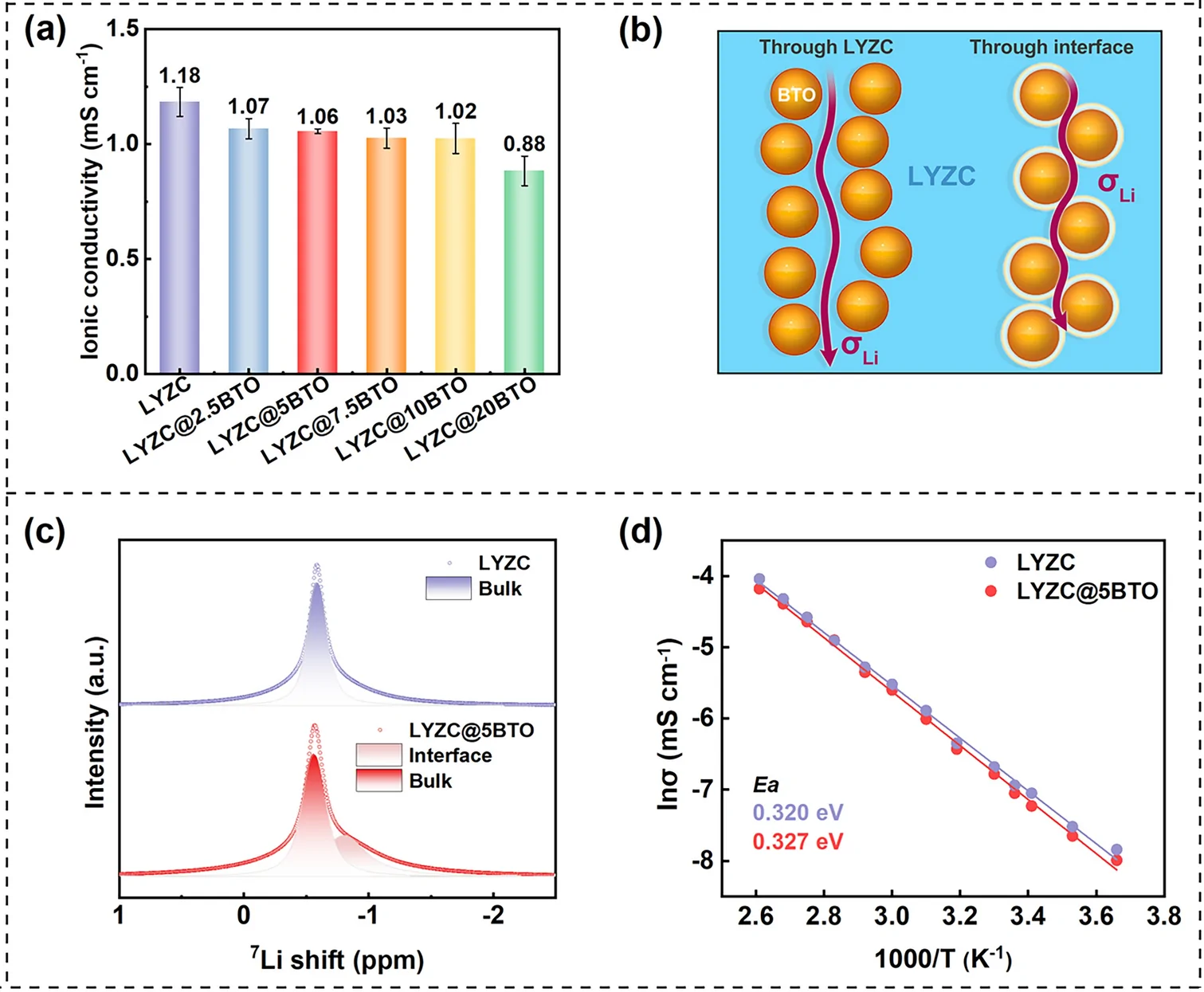
**a** Ion conductivity of LYZC@*x*BTO (*x* = 0, 2.5, 5, 7.5, 10, 20) at 25 °C (bar chart). **b** Schematic illustration of two possible Li^+^ conduction pathways in LYZC@BTO composite electrolytes. **c** ^7^Li SS-NMR spectra of pristine LYZC and LYZC@5BTO. **d** Arrhenius plots of ionic conductivity for LYZC and LYZC@5BTO

Although tetragonal BTO is intrinsically inert with respect to Li^+^ conduction, its nanostructured form may enable unconventional Li^+^ transport mechanisms that go beyond conventional bulk diffusion. Therefore, it is likely to exert a relatively minor influence on the ionic conductivity of the LYZC electrolyte. To investigate this hypothesis, electrochemical impedance spectroscopy (EIS) was conducted on LYZC@*x*BTO composite electrolytes with varying BTO contents (*x* = 0, 2.5, 5, 7.5, 10, and 20 wt.%) at 25 °C (Fig. 3a). The Nyquist plots and pellet thicknesses shown in Fig. S7a, b were used to calculate the corresponding ionic conductivities. Error bars represent the standard deviations obtained from three independent samples, which were included to ensure statistical reliability and reproducibility. As depicted in Fig. 3a, BTO loadings ≤ 5 wt.% do not significantly diminish the ionic conductivity compared to pristine LYZC, while a 20 wt.% BTO content results in a ~ 33% reduction. These results indicate that the introduction of ionically inert BTO nanoparticles has a negligible impact on bulk conduction within an optimal concentration range. As illustrated in Fig. 3b, we attribute this behavior to enhanced surface-mediated Li^+^ conduction at the LYZC/BTO interfaces, enabled by interfacial interactions between the LYZC electrolyte matrix and the dispersed BTO nanoparticles. This mechanism is reminiscent of those observed in polymer–ceramic composite electrolytes [42, 43]. Our SSNMR analysis, as shown in Fig. 3c, supports this hypothesis. The LYZC@5BTO spectrum shows a shoulder peak at -0.5 ppm, which is indicative of Li⁺ diffusion along the surface of the LYZC particles. This finding is in agreement with the observations made by Kwak et al. [44]. Activation energy calculations (Figs. 3d and S8) give values of 0.320 eV (LYZC) and 0.327 eV (LYZC@5BTO), showing minimal disruption to bulk Li^+^ conduction paths. The results confirm that BTO coating enhances surface conduction of LYZC electrolyte without affecting its bulk properties. DC polarization measurements (Fig. S9) show an electronic conductivity of 5.4 × 10^–9^ S cm^−1^ for LYZC@5BTO, similar to pristine LYZC and 6-order of magnitude lower than its ionic conductivity, confirming LYZC@5BTO as a suitable Li^+^-selective conductor for ASSBs [45].

### Electrochemical Performance of ASSBs Based on LYZC@BTO

**Fig. 4:**
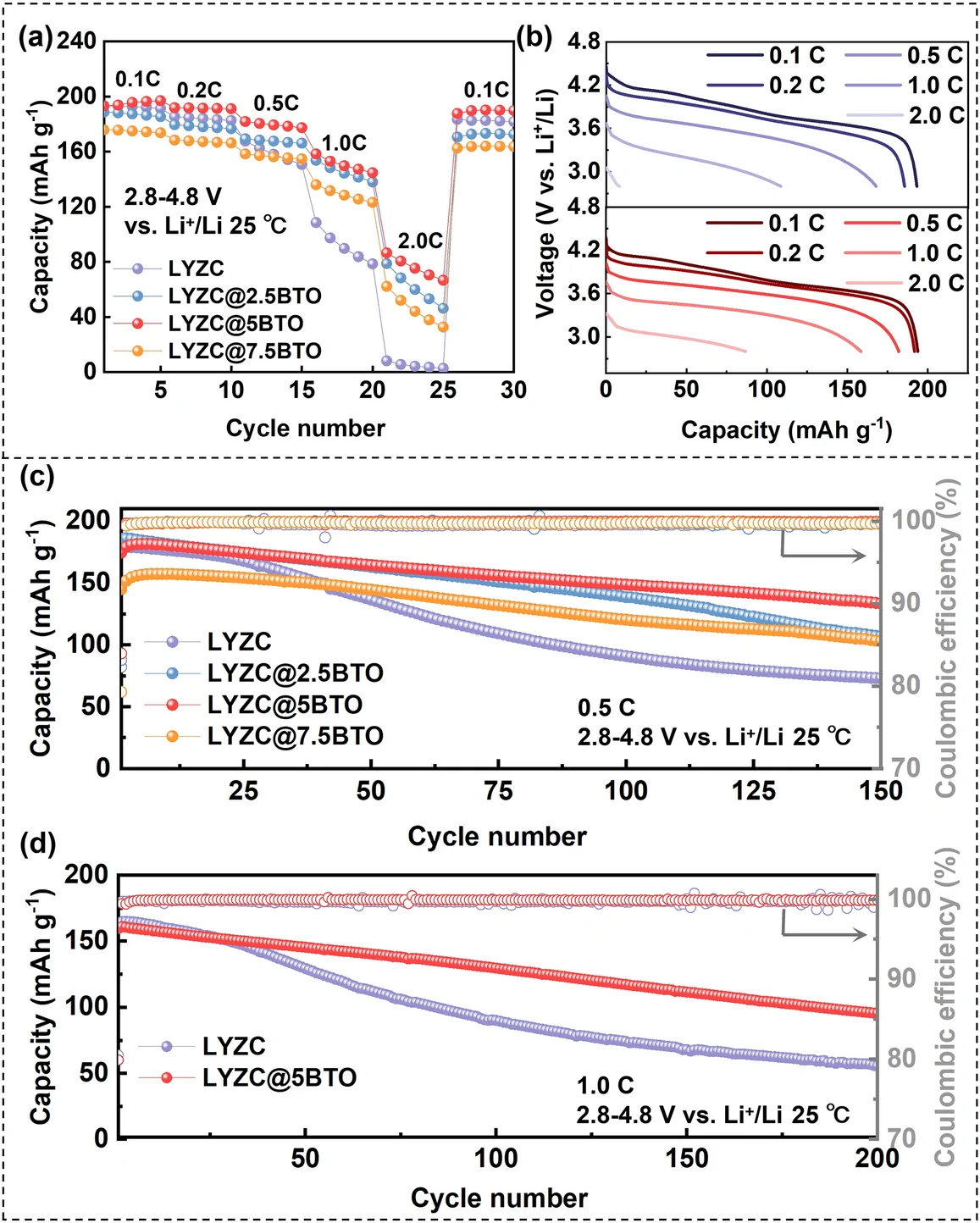
**a** Rate performance of LYZC@*x*BTO (*x* = 0, 2.5, 5, and 7.5) within the voltage range of 2.8–4.8 V vs. Li^+^/Li and **b** corresponding discharge profiles of LYZC and LYZC@5BTO. **c** Cycling performance of LYZC@*x*BTO (*x* = 0, 2.5, 5, and 7.5) at 0.5 C within the voltage range of 2.8–4.8 V vs. Li^+^/Li. **d** Cycling performance of LYZC and LYZC@5BTO at 1.0 C within the voltage range of 2.8–4.8 V vs. Li^+^/Li

To investigate the impact of ferroelectric BTO coatings on electrolyte electrochemical performance at high voltages, ASSBs with the configuration LiIn|LYZC@*x*BTO (*x* = 0, 2.5, 5, 7.5)*|*LYZC*@x*BTO-SCNCM811 (3:7 mass ratio) were tested in the voltage ranges of 2.8–4.3 and 2.8–4.8 V versus Li^+^/Li at room temperature. As shown in Fig. S10, the cycling performance of LYZC and LYZC@5BTO at 1 C (within the 2.8–4.3 V range vs. Li^+^/Li) reveals negligible differences in performance: Both pristine LYZC and LYZC@5BTO exhibit nearly identical capacity retentions (58.0% vs. 63.7% after 200 cycles). This indicates that the field-modulating effect of BTO is insignificant below the decomposition threshold of LYZC (~ 4.3 V). Thus, we evaluated the cell performance at a cut-off voltage of 4.8 V. As depicted in Fig. 4a, ASSBs with LYZC@5BTO deliver the highest initial discharge capacity of 193.2 mAh g^−1^ at 0.1 C (1 C = 200 mA g^−1^), compared to 193.1 mAh g^−1^ for pristine LYZC. At higher rates of 0.2, 0.5, 1.0, and 2.0 C, ASSBs with LYZC@5BTO maintained superior capacities of 191.9, 181.9, 158.4, and 86.5 mAh g^−1^, respectively, versus 185.5, 167.7, 108.5, and 8.2 mAh g^−1^ for pristine LYZC. The better rate performance is due to the enhanced high-voltage stability of LYZC@5BTO, which exhibits smaller polarization voltage than pristine LYZC (Fig. 4b). It is speculated that the coating layer may be discontinuous for LYZC@2.5BTO, thereby failing to completely suppress interfacial reactions. Additionally, the relatively low ionic conductivity of LYZC@7.5BTO and the thick coating layer may hinder ionic and/or electronic transport [46].

Long-term performance of ASSBs with pristine LYZC and LYZC@*x*BTO (*x* = 2.5, 5, 7.5) at 0.5 C was evaluated, shown in Fig. 4c. LiIn|LYZC@5BTO|LYZC@5BTO-SCNCM811 cells displayed enhanced cyclic stability at 0.5 C, with an initial discharge capacity of 174.6 mAh g^−1^, retaining 76% capacity after 150 cycles, surpassing other tested samples. At 1.0 C (Figs. 4d and S11), LYZC@5BTO delivered 95.4 mAh g^−1^ after 200 cycles, nearly double that of pristine LYZC. When the cutoff voltage was further elevated to 5 V, the LYZC@5BTO cell still maintained a capacity retention of 79.8% after 100 cycles (Fig. S12). These findings confirm that the BTO surface coating improves electrochemical performance of LYZC at high voltages. Differential capacity (dQ/dV) curves (Fig. S13) elucidate changes in voltage plateaus for LYZC and LYZC@5BTO at 1.0 C. For LYZC@5BTO, the initial anodic/cathodic peaks at 3.86/3.63 V (1st cycle) shift to 3.93/3.43 V after 100 cycles, widening the potential interval from 0.23 to 0.50 V. In contrast, pristine LYZC exhibits a more pronounced increase in the potential interval, expanding from 0.27 to 0.81 V under identical conditions, indicating higher polarization [47]. After 200 cycles, LYZC@5BTO’s potential interval remained 24%, which is smaller than LYZC’s. These findings collectively confirm that ferroelectric BTO coatings mitigate polarization in ASSBs, thereby enhancing their stability during high-voltage cycling.

### Electric Field Modulation and High-Voltage Resistance Capability of LYZC@BTO

**Fig. 5:**
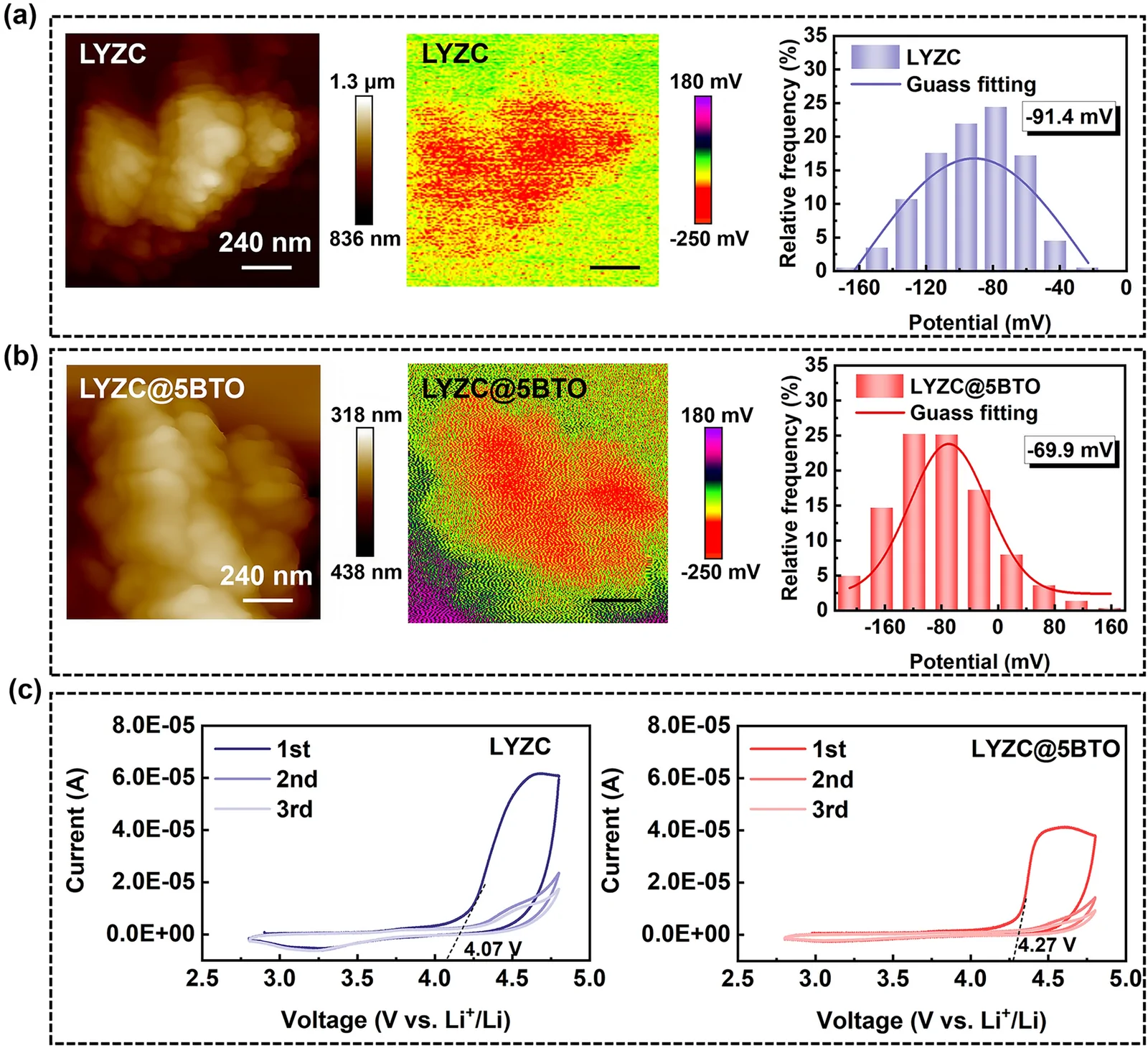
AFM images of KPFM and LYZC@5BTO, with surface potential maps of the specific regions for **a** LYZC and **b** LYZC@5BTO. CV plots of **c** LYZC and LYZC@5BTO cells

To understand the mechanisms of improved high-voltage battery stability, we investigated two key aspects: the intrinsic high-voltage resistance of the electrolyte and its compatibility with high-voltage cathode materials. Given the potential of ferroelectric BTO’s electric field-modulating capability to influence electrolyte decomposition at high voltages, we first employed Kelvin probe force microscopy (KPFM) to characterize surface potential changes in LYZC before and after BTO modification. As shown in Fig. 5a, b, the average potential for LYZC and LYZC@5BTO were -91.4 and -69.9 mV, respectively, suggesting that LYZC@5BTO exhibits a lower absolute surface potential compared to pristine LYZC. This indicates that BTO’s polarization reversal induces an internal electric field at the LYZC/BTO interface [48], which counteracts the external electric field, thereby reducing the effective electric field experienced by the electrolyte. This mechanism suppresses voltage-driven electrolyte self-decomposition, enhancing battery stability. To further validate this hypothesis, cyclic voltammetry (CV) was used to evaluate electrolyte oxidation behavior at high voltages. Model cells with the configuration LiIn|LYZC@*x*BTO (*x* = 0, 5)|LYZC@*x*BTO-C (9:1 mass ratio) were tested between 2.8 and 4.8 V and Li^+^/Li. For pristine LYZC (Fig. 5c), an intense anodic peak at 4.07 V (vs. Li^+^/Li) appeared in the first CV cycle, shifting to 4.25 V with reduced peak area in subsequent cycles, indicating progressive electrolyte decomposition, which is consistent with the behavior of LIC electrolytes reported by Deng et al*.* [49]. In contrast, the LYZC@5BTO cell exhibited higher anodic peaks at 4.27 V (first cycle) and 4.5 V (subsequent cycles), with substantially smaller oxidation peak areas, confirming that BTO’s electric field modulation suppresses LYZC decomposition under high-voltage conditions.

### Mechanisms of Interfacial and Structural Evolution of SCNCM811/LYZC@BTO

**Fig. 6:**
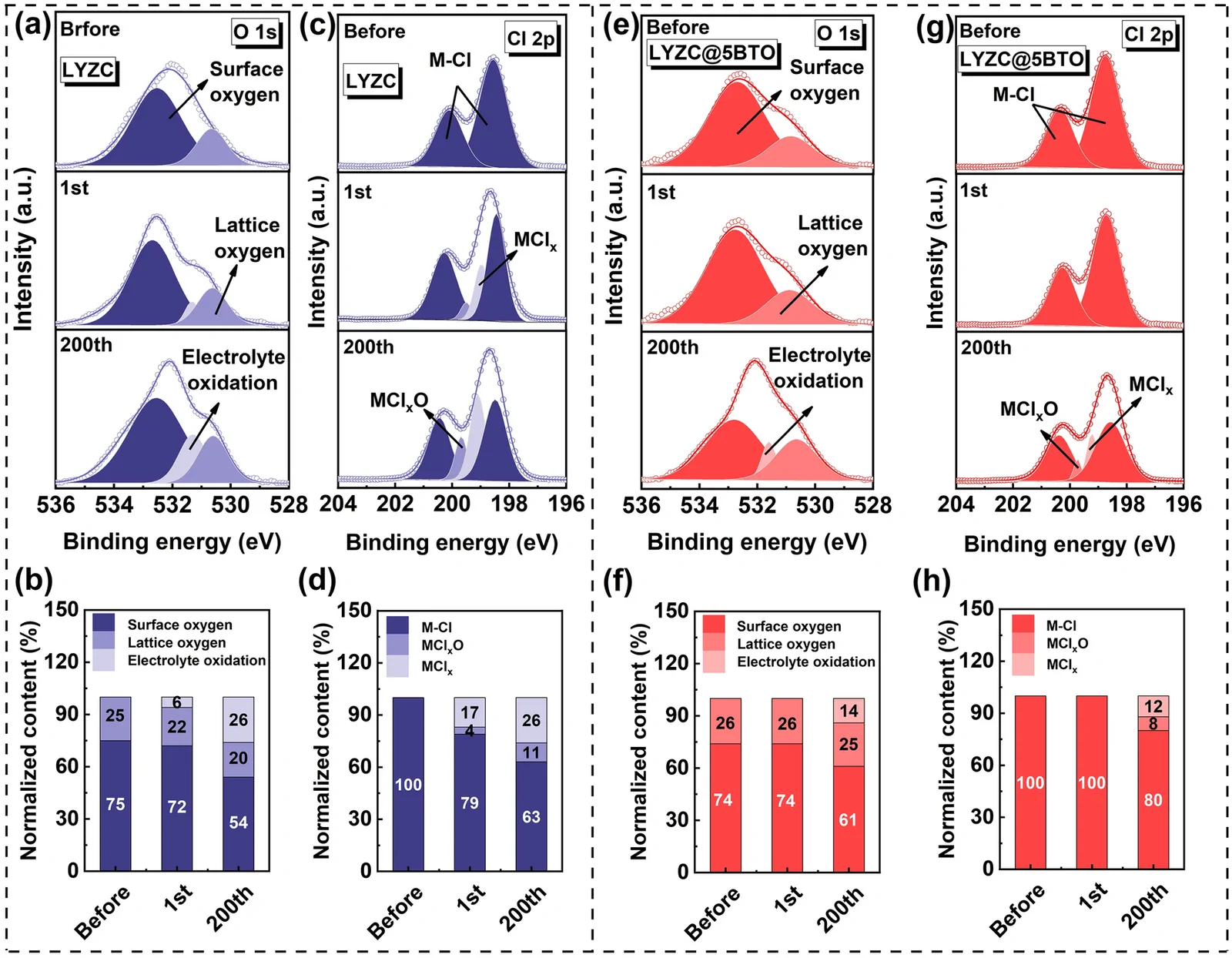
XPS evaluation on the SCNCM811/CSEs interface on the LiIn||SCNCM811 cells before and after cycling. **a, b** O 1*s* and **c, d** Cl 2*p* XPS spectra of LYZC with the corresponding compositional analysis in the pristine, 1st cycle discharged and 200th cycle discharged states. **e, f** O 1*s* and **g, h** Cl 2*p* XPS spectra of LYZC@5BTO with the corresponding compositional analysis in the pristine, 1st cycle discharged and 200th cycle discharged states

X-ray photoelectron spectroscopy (XPS) was employed to characterize surface property evolution at the SCNCM811/CSEs interface during cycling. For pristine LYZC, the O 2*p* spectrum (Fig. 6a) exhibits two peaks at 532.4 eV (surface-adsorbed oxygen) and 530.5 eV (lattice oxygen in SCNCM811) [50]. After the first cycle, a new peak emerges at 532.0 eV, assigned to Metal-O-Cl species (e.g., Zr/Y–O–Cl) [51], indicating interfacial decomposition between LYZC and SCNCM811. Quantification (Fig. 6b) shows Metal-O-Cl compounds account for 6% of surface species initially, increasing to 26% after 200 cycles due to cumulative side reactions. The Cl 2*p* spectra of LYZC (Fig. 6c) follow a similar trend: in addition to the characteristic chloride electrolyte doublets (201/199 eV) [52], minor peaks at 199.5 eV (Metal-O-Cl) and 199.0 eV (Metal-Cl, e.g., LiCl) appear after the first cycle [53]. After 200 cycles, their proportions rose from 4 and 17% to 11% and 26%, respectively (Fig. 6d), confirming progressive LYZC decomposition and by-product accumulation. In contrast, LYZC@5BTO (Fig. 6e-h) shows significantly reduced by-products: Metal-O-Cl and Metal-Cl contents remain 0% after the first cycle and increase only to 8% and 12% after 200 cycles. These results conclusively demonstrate that ferroelectric BTO coating via ball-milling suppresses the formation of interfacial decomposition products (e.g., Zr/Y–O-Cl, LiCl/ZrCl_4_), thereby stabilizing the SCNCM811/CSE interface during high-voltage cycling.

**Fig. 7:**
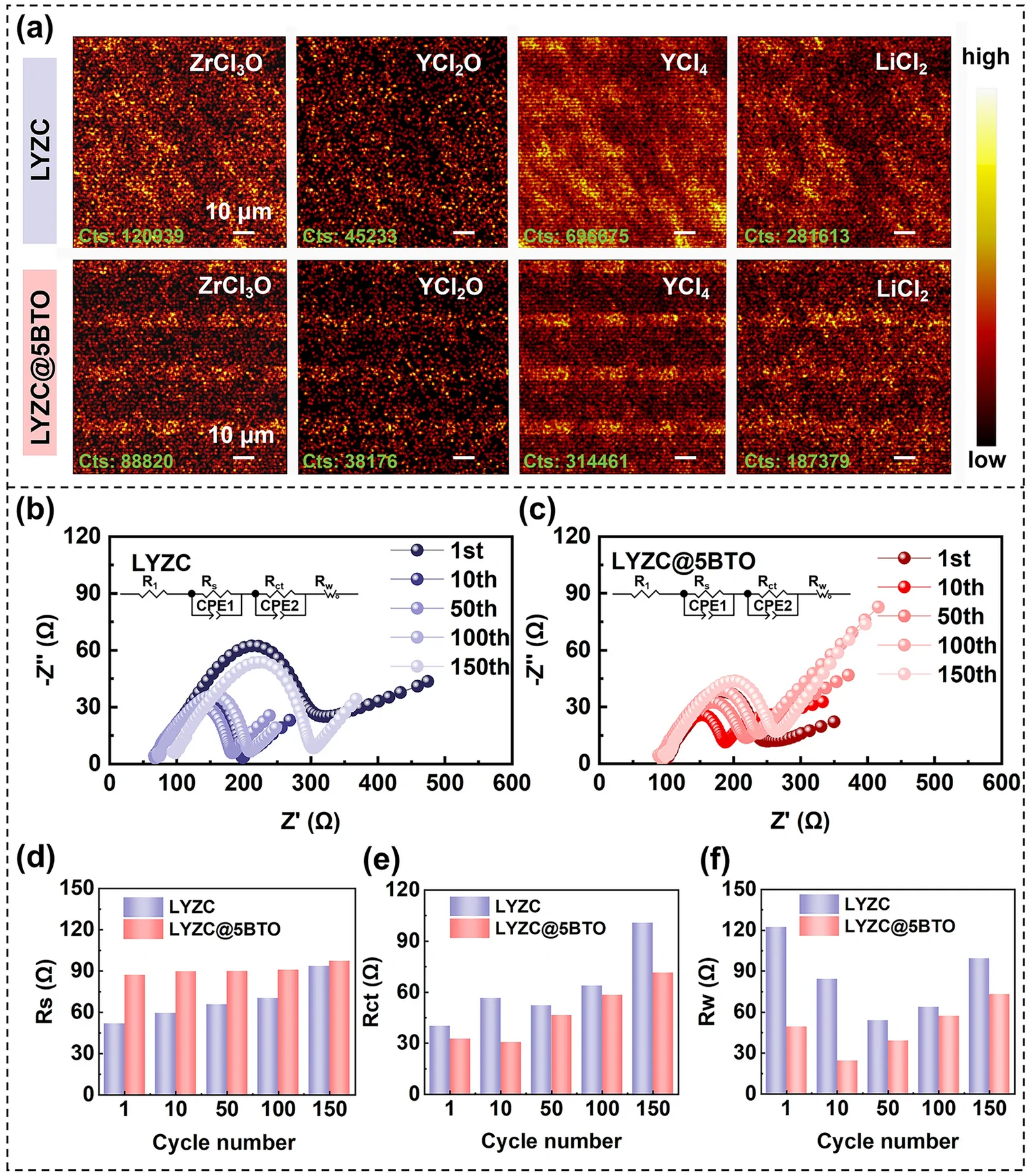
**a** ToF–SIMS images of cycled SCNCM811/LYZC composite layer and SCNCM811/LYZC@5BTO composite layer, with counts (Cts) of ionic fragment groups included. The Nyquist plots of **b** LYZC and **c** LYZC@5BTO ASSBs at different cycle numbers, with insets showing the fitted equivalent circuits. **d-f** Fitting results of the Nyquist plots for LYZC and LYZC@5BTO cells at different cycle number

To comprehensively investigate the chemical and electrochemical changes at the SCNCM811/CSEs interface, time-of-flight secondary ion mass spectrometry (ToF–SIMS) and electrochemical impedance spectroscopy (EIS) were employed. As depicted in Fig. 7, the counts (Cts) of ionic fragment groups (ZrCl_3_O, YCl_2_O, YCl_4_, and LiCl_2_) within the SCNCM811/LYZC@5BTO composite layer exhibit lower signal intensities than those in the SCNCM811/LYZC composite layer (88,820 vs. 120,939 for ZrCl_3_O, 38,176 vs. 45,233 for YCl_2_O, 31,4461 vs. 696,675 for YCl_4_, and 187,379 vs. 281,613 for LiCl_2_). In agreement with the XPS findings in Fig. 6, ToF–SIMS analysis indicates that cycled SCNCM811/LYZC@5BTO composites demonstrate substantially reduced surface concentrations of decomposition by-products (ZrCl_3_O and YCl_2_O) relative to the SCNCM811/LYZC composites. Furthermore, the incorporation of 5 wt.% BTO effectively mitigates the decomposition of the LYZC electrolyte, as evidenced by the decreased concentrations of YCl_4_ and LiCl_2_ species (Fig. 7a).

EIS measurements conducted on LiIn|LYZC|LYZC-SCNCM811 and LiIn|LYZC@5BTO|LYZC@5BTO-SCNCM811 ASSBs at various cycle numbers under 1.0 C (Fig. 7b, c, with insets showing fitted equivalent circuits) to provide deeper insights into their electrochemical behaviors. Typically, the high-frequency region corresponds to intrinsic resistance (*R*_s_), the mid-frequency region reflects electrode–electrolyte interfacial impedance (*R*_ct_), and the diagonal line in the Nyquist plots represents Li^+^ diffusion impedance (*R*_w_) in the electrode [54]. As illustrated by the fitting results in Fig. 7d-f, LYZC@5BTO exhibits higher *R*_s_ values compared to pristine LYZC owing to its slightly lower ionic conductivity; however, during cycling, *R*_s_ remains stable for LYZC@5BTO while steadily increasing for pristine LYZC, indicating severe progressive structural degradation in the latter. Furthermore, LYZC@5BTO consistently demonstrates lower *R*_ct_ values across all cycles, suggesting effective suppression of the accumulation of surface side reaction products [55, 56]. For *R*_w_, both samples exhibit a decreasing trend in diffusion impedance over the first 50 cycles, followed by an increase. This phenomenon stems from electrochemical-mechanical coupling during cycling: in the initial 50 cycles, cathode activation enhances Li^+^ transport and reduces diffusion impedance; later, oxygen loss induces structural degradation, leading to increased impedance. LYZC@5BTO displays slower *R*_w_ growth due to BTO’s electric field homogenization effect. The ToF–SIMS and EIS findings further confirm that the ferroelectric BTO coating facilitates directional Li^+^ migration, mitigates interfacial Li^+^ accumulation, and maintains unobstructed Li^+^ transport pathways across the SCNCM811/CSE interface, thereby enhancing cycling stability under high-voltage conditions [57].

**Fig. 8:**
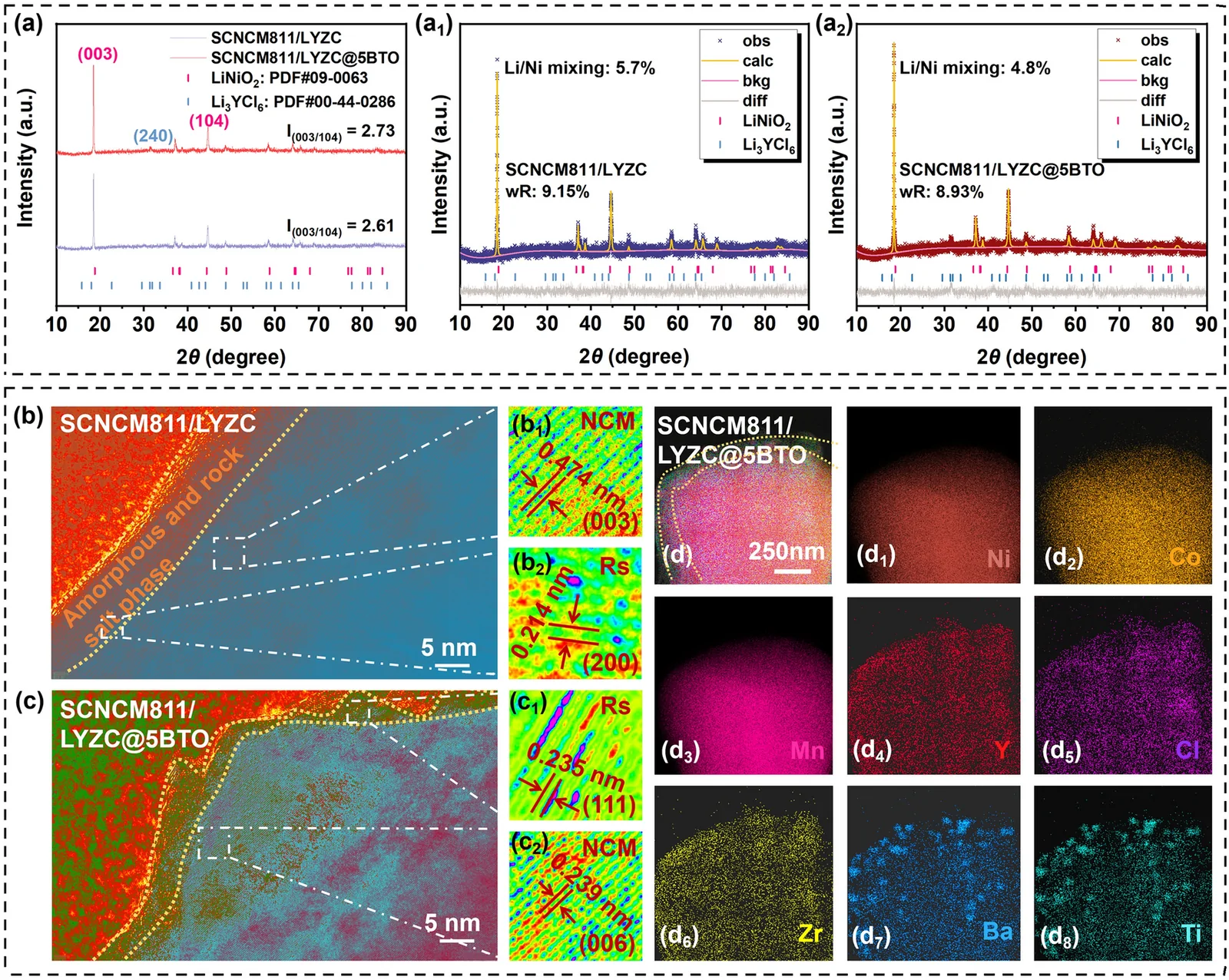
**a** XRD patterns and corresponding Rietveld refinement of **a**_**1**_ SCNCM811/LYZC composite and **a**_**2**_ SCNCM811/LYZC@5BTO composite after 200 cycles. **b** High-resolution TEM image of SCNCM811/LYZC composite after 200 cycles with **b**_**1**_ lattice plane image of SCNCM-(003) and **b**_**2**_ Rock-salt (Rs)-(200). **c** High-resolution TEM image of SCNCM811/LYZC@5BTO composite after 200 cycles with **c**_**1**_ lattice plane images of Rs-(002), **c**_**2**_ SCNCM-(006). **d** TEM images of SCNCM811/LYZC composite after 200 cycles with corresponding EDS elemental mappings of **d**_**1**_-**d**_**8**_ Ni, Co, Mn, Y, Cl, Zr, Ba, and Ti, respectively

We further employed XRD and HRTEM to investigate the structural changes of SCNCM811/CSEs composites after cycling. Figure 8a presents the XRD patterns and Rietveld refinement results of the cycled SCNCM811/LYZC and SCNCM811/LYZC@5BTO, with the refined crystallographic data listed in Tables S2 and S3. Due to the relatively low electrolyte content within the composite layer, the characteristic peaks of LYZC are barely observable. The intensity ratio of the (003) to (104) peaks indicates that, after cycling, the SCNCM811 in SCNCM811/LYZC@5BTO (2.73) retains a more distinct layered structure compared to that in SCNCM811/LYZC (2.61) [58]. Refinement results further demonstrate that the degree of Li/Ni mixing in the cathode of SCNCM811/LYZC@5BTO is 0.9% lower than that in SCNCM811/LYZC. Typically, the increase in Li/Ni cation mixing after cycling is attributed to the formation of oxygen vacancies, which lower the energy barrier for Ni migration into Li sites [59]. These findings indicate that fewer oxygen vacancies are generated in NCM811 of SCNCM811/LYZC@5BTO during high-voltage cycling, thereby helping preserve the layered structure and facilitating Li-ion intercalation and deintercalation.

HRTEM analysis indicates that the nanostructure of cycled SCNCM811/LYZC particles consists of an amorphous phase and a rock-salt phase (with a thickness of ~ 10 nm), and a layered structure (Figs. 8b and S14). This amorphous phase may be an interfacial by-product, while the rock-salt phase could originate from the transformation of the layered structure [60]. Such structural changes are likely initiated by surface oxygen loss, which arises from side reactions between LYZC and NCM [61]. In contrast, SCNCM811/LYZC@5BTO retains a relatively stable layered phase, with the thickness of the rock-salt phase remaining below ~ 5 nm. This suggests that SCNCM811 with the LYZC@5BTO electrolyte undergoes fewer phase transformations during cycling, consistent with the XRD analysis results. Furthermore, as shown in the EDS mapping results in Fig. 8d, the LYZC@5BTO layer is approximately 100 nm thick and uniformly coats the surface of the NCM811 cathode. These observations suggest that BTO functions as a protective layer, effectively reducing surface-induced side reactions and suppressing oxygen loss caused by irreversible phase transitions. The mechanism underlying BTO-induced degradation suppression can be attributed to two key factors: electric field homogenization, which minimizes localized oxygen release, and Li^+^ flux regulation, which promotes uniform lithiation/delithiation kinetics. SEM analysis of the cycled SCNCM811/LYZC and SCNCM811/LYZC@5BTO composites (Fig. S15) supports these conclusions. While the SCNCM811/LYZC composite exhibits significant cracking after high-voltage cycling, which disrupts the Li^+^ transport network within the cathode, the SCNCM811/LYZC@5BTO composite maintains excellent morphological stability even after 200 cycles.

**Fig. 9:**
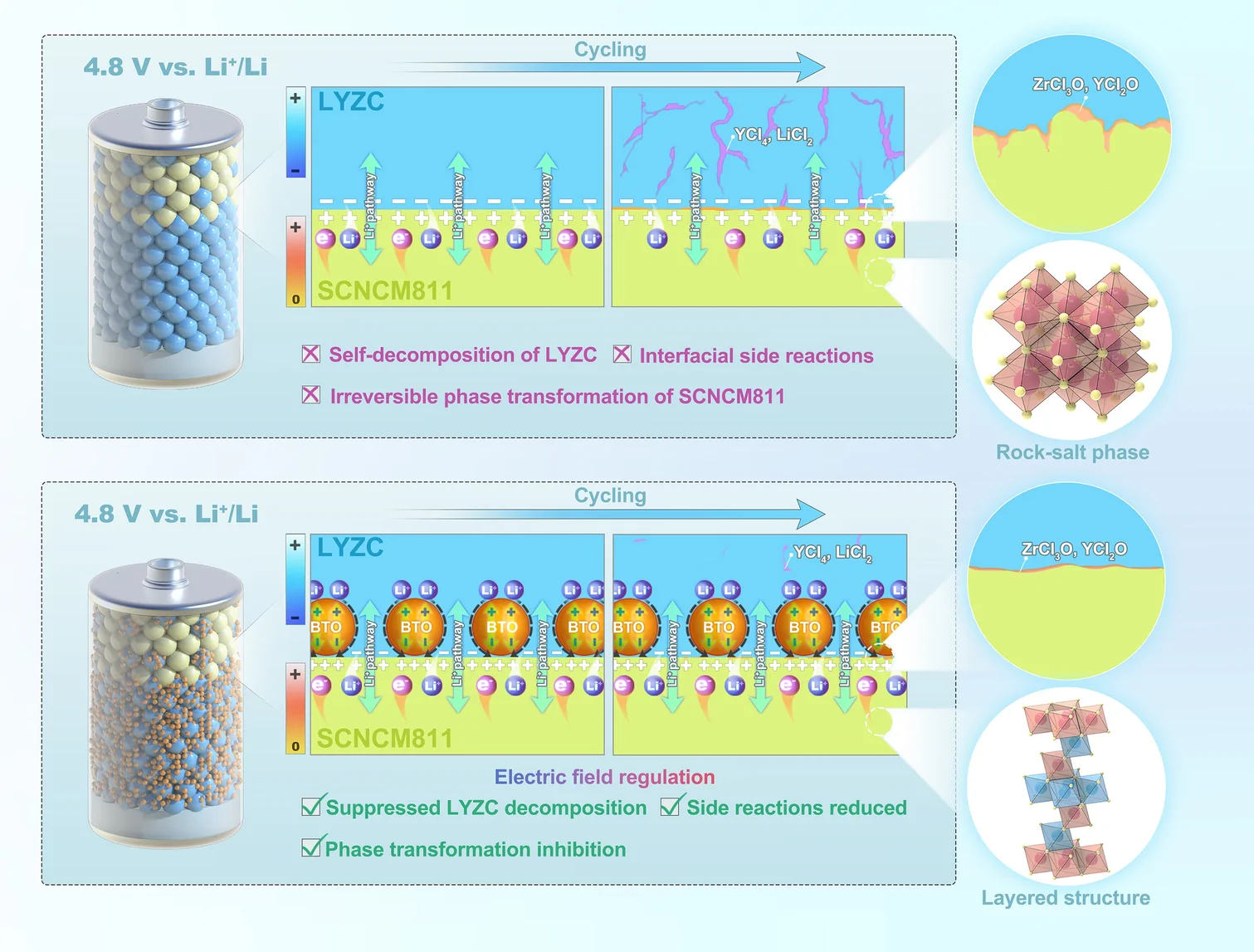
Schematic diagram demonstrating the advantages of utilizing ferroelectric nano-BTO particles to regulate the interfacial electric field of LYZC electrolyte and improve the stability of electrolyte–electrode interfaces in high-voltage (4.8 V) ASSBs

Findings from ToF–SIMS, EIS, XRD, HRTEM, and SEM analyses indicate that when LYZC is coupled with SCNCM811 under high-voltage cycling conditions (4.8 V vs. Li^+^/Li), it is susceptible to self-decomposition, yielding products such as YCl_4_ and LiCl_2_. Simultaneously, interfacial side reactions are likely to occur at the LYZC/SCNCM811 interface, resulting in the formation of by-products including ZrCl_3_O and YCl_2_O. These by-products exhibit electrochemical inertness toward Li^+^ ions, thereby obstructing their efficient transport; their progressive accumulation contributes to elevated electrolyte and interfacial impedance. Furthermore, oxygen loss may induce an irreversible surface phase transition from a layered structure to a rock-salt structure in the cathode material, compromising its structural integrity and impeding bulk Li^+^ diffusion. Collectively, these detrimental processes lead to capacity fading in LYZC-based ASSBs under high-voltage cycling. As illustrated in Fig. 9, the optimization of the interfacial electric field through the incorporation of ferroelectric BTO nanoparticles effectively mitigates voltage-induced self-decomposition of chloride-based solid electrolytes, suppresses detrimental interfacial reactions with layered oxide cathodes, and inhibits the occurrence of irreversible phase transitions in SCNCM811. Consequently, this approach significantly improves the cycling stability of chloride electrolytes under high-voltage conditions, facilitating stable operation at voltages up to 4.8 V in ASSBs.

## Conclusions

We propose an interfacial electric field modulation strategy by incorporating ferroelectric BTO nanoparticles into commercially available LYZC chloride electrolytes to enhance their oxidation resistance under ultrahigh-voltage conditions (4.8 V). The addition of 5 wt.% ionically insulating BTO (LYZC@5BTO) maintains high Li^+^ conductivity, with only a slight decrease from 1.18 to 1.06 mS cm^−1^. The intrinsic polarization of BTO induces an internal electric field at the LYZC/BTO interface, which offsets the external high-voltage field and suppresses electrolyte self-decomposition. Moreover, the presence of BTO significantly mitigates interfacial side reactions between LYZC and the layered cathode SCNCM811 during high-voltage cycling. This results in a marked reduction in parasitic by-products such as ZrCl_3_O and YCl_2_O, thereby improving interfacial Li^+^ transport kinetics during cycling. Moreover, the irreversible surface phase transition from layered structure to rock-salt structure in the SCNCM811 cathode material is effectively suppressed. Consequently, all-solid-state batteries employing LYZC@5BTO achieve a high specific capacity of 95.4 mAh g^−1^ after 200 cycles at 1 C under 4.8 V, compared to only 55.4 mAh g^−1^ for unmodified LYZC. This work demonstrates a scalable, cost-effective surface modification approach and introduces a novel electric field regulation mechanism, offering a promising pathway toward the development of high-voltage-resistant chloride solid electrolytes for next-generation high-energy–density ASSBs.

## Supplementary Information

Below is the link to the electronic supplementary material.

Supplementary file1 (DOCX 36246 KB)
