# Supplementary material for: BaTiO3 Nanoparticle-Induced Interfacial Electric Field Optimization in Chloride Solid Electrolytes for 4.8 V All-Solid-State Lithium Batteries
Source: Nanomicro Lett. 2025 Sep 1;18:52. doi: 10.1007/s40820-025-01901-2 (PMC12401864; doi:10.1007/s40820-025-01901-2)
Supplement: Supplementary file 1 — Supplementary file1 (DOCX 36246 KB) [file 40820_2025_1901_MOESM1_ESM.docx]

Supporting Information for

**BaTiO_3_ Nanoparticle-Induced Interfacial Electric Field Optimization in Chloride Solid Electrolytes for 4.8 V All-Solid-State Lithium Batteries**

Qingmei Xiao^1^, Shiming Huang^1^, Donghao Liang^1^, Cheng Liu^1^, Ruonan Zhang^1^, Wenjin Li^1^*, Guangliang Gary Liu^1^*

^1^Guangdong Provincial Key Laboratory of New Energy Materials Service Safety, College of Materials Science and Engineering, Shenzhen University, Shenzhen 518060, P. R. China

*Corresponding authors. E-mails: [liwj@szu.edu.cn](mailto:liwj@szu.edu.cn) (Wenjin Li); [ggliu@szu.edu.cn](mailto:ggliu@szu.edu.cn) (Guangliang Gary Liu)

**Supplementary Figures and Tables**


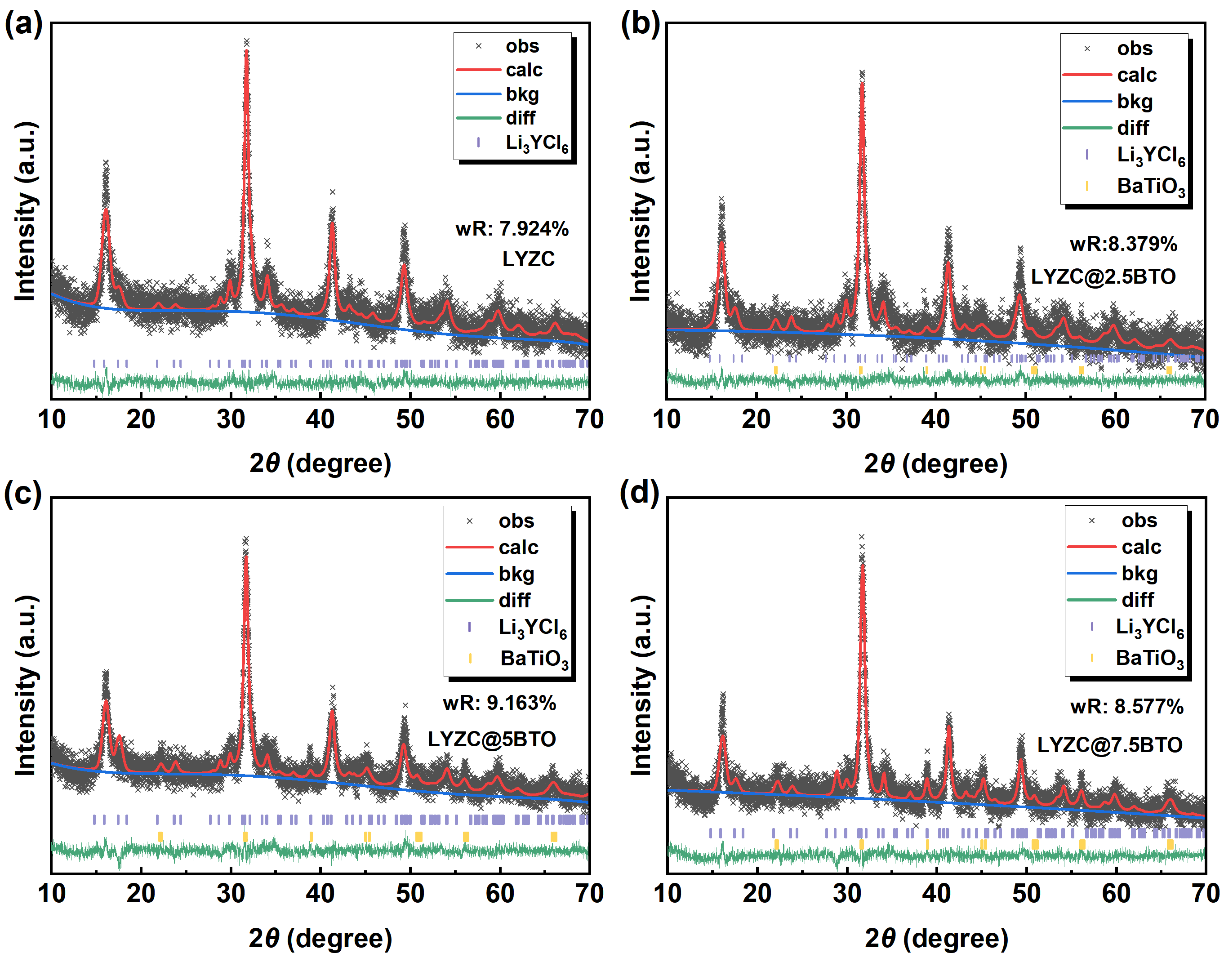


**Fig. S1** The Rietveld refinement of LYZC@*x*BTO **(a)** *x* = 0, **(b)** *x* = 2.5, **(c)** *x* = 5, and **(d)** *x* = 7.5





**Fig. S2** XPS characterization of Ti 2p, Ba 3d for **(a, b)** LYZC and **(c, d)** LYZC@5BTO





**Fig. S3** The XPS characterization of **(a)** Y 3d, **(b)** Zr 3d, and **(c)** Cl 2p for LYZC and LYZC@5BTO


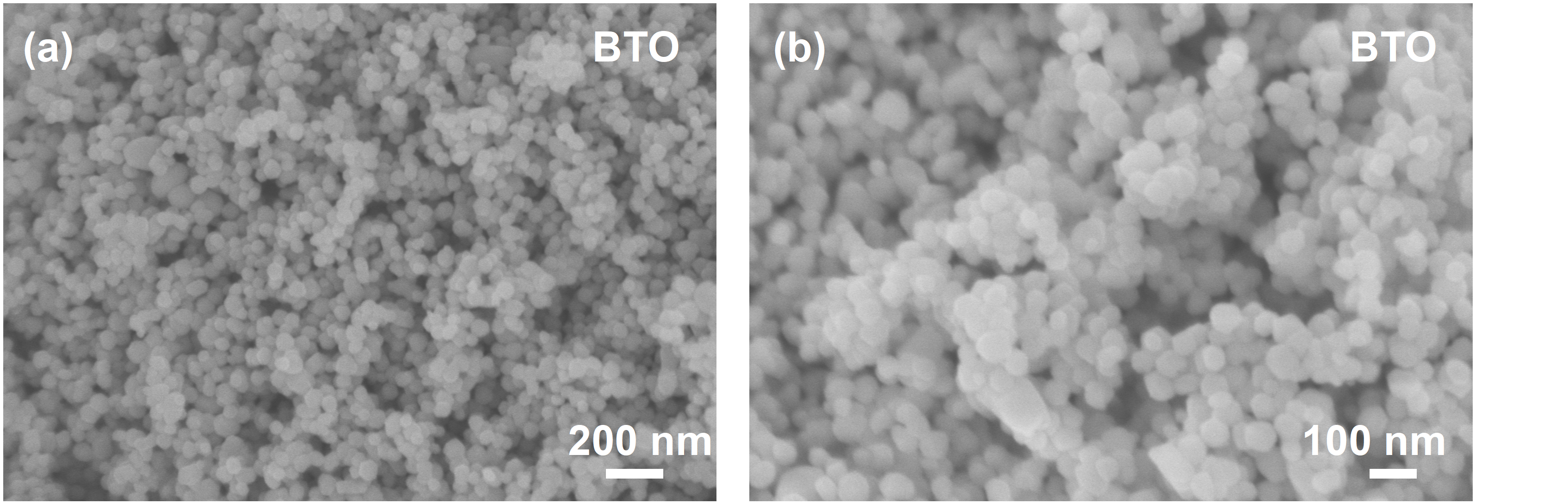


**Fig. S4** **(a, b)** SEM images of nano BTO particles


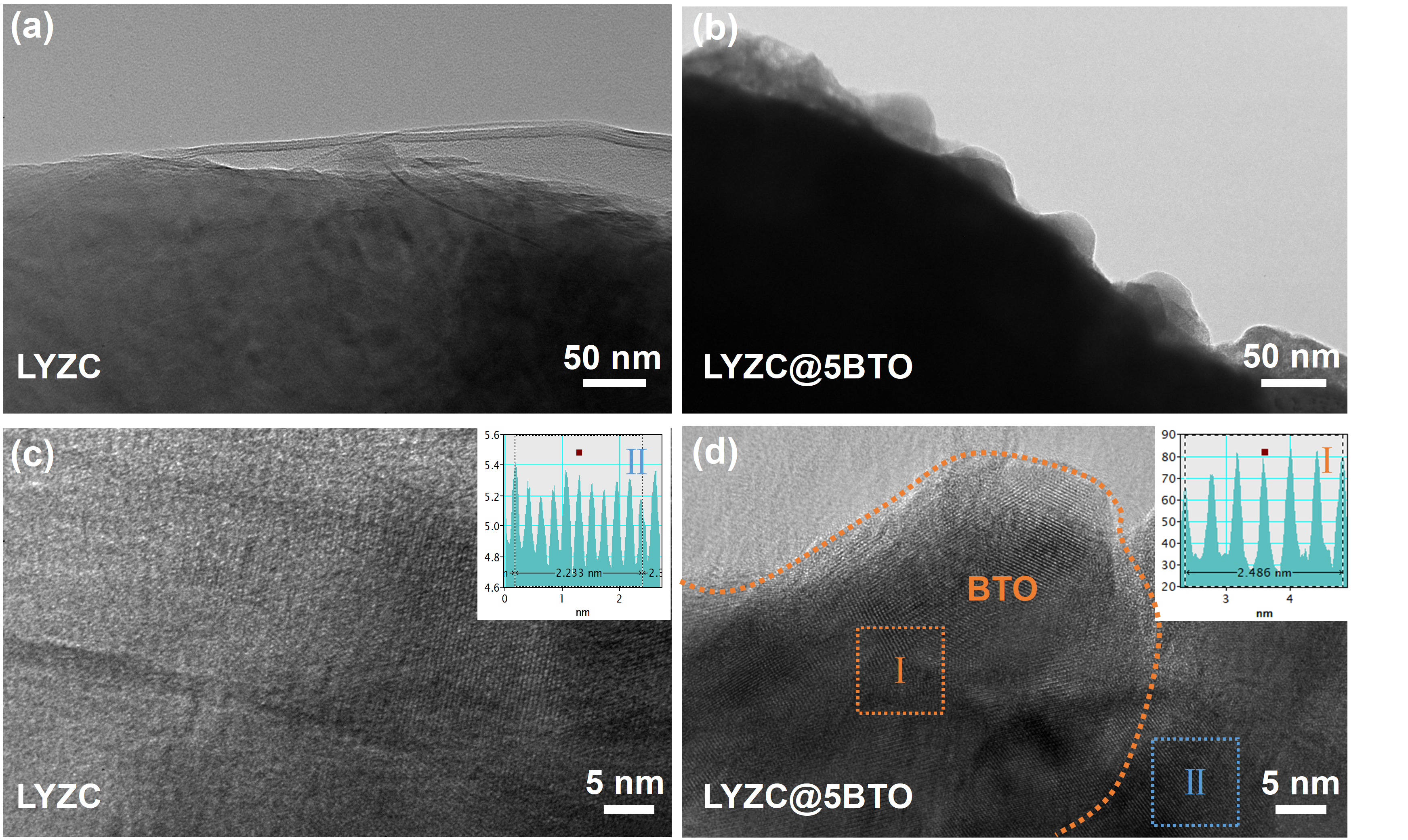


**Fig. S5** The TEM image of **(a)** LYZC and **(b)** LYZC@5BTO. High-resolution TEM image of **(c)** LYZC and **(d)** LYZC@5BTO. In Figure (d), Region I corresponds to the morphology of BTO, while Region Ⅱ corresponds to LYZC


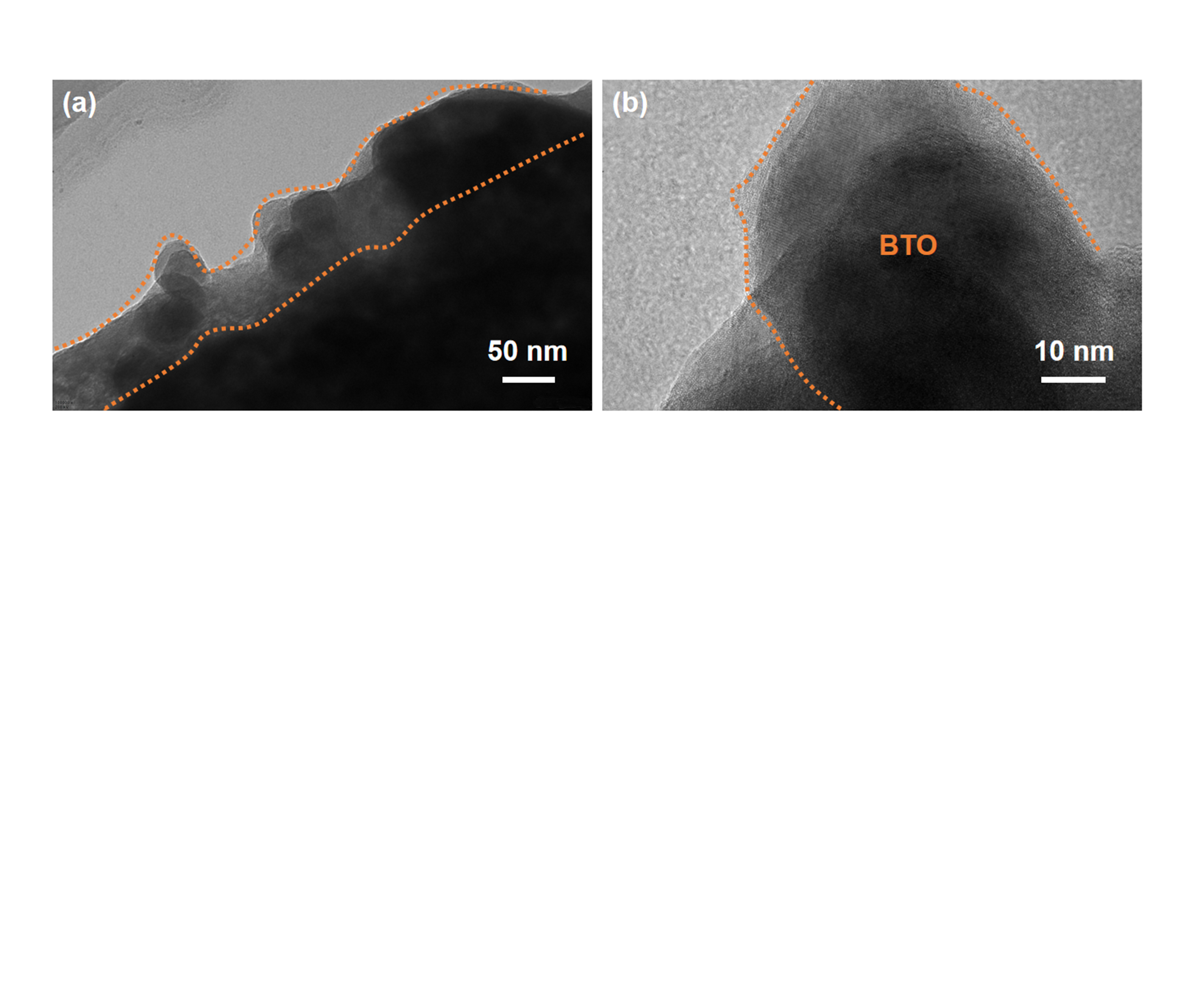


**Fig. S6 (a, b)** The TEM images of LYZC@5BTO


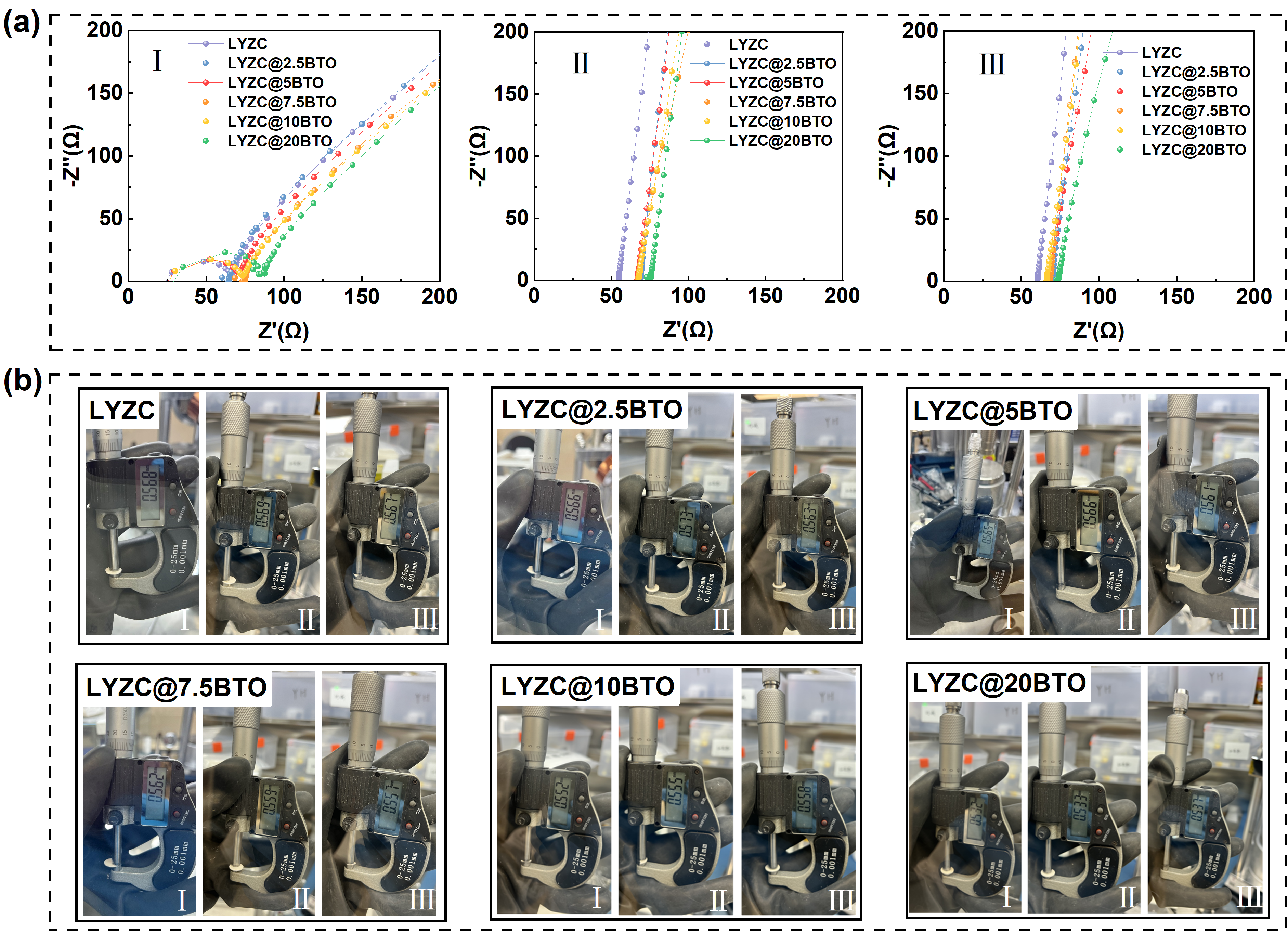


**Fig. S7** **(a)** The Nyquist plots of LYZC@*x*BTO (*x* = 0, 2.5, 5, 7.5, 10, 20) measured at 25 ℃. **(b)** Schematic diagram of measured thickness for LYZC@*x*BTO (*x* = 0, 2.5, 5, 7.5, 10, 20) pellets


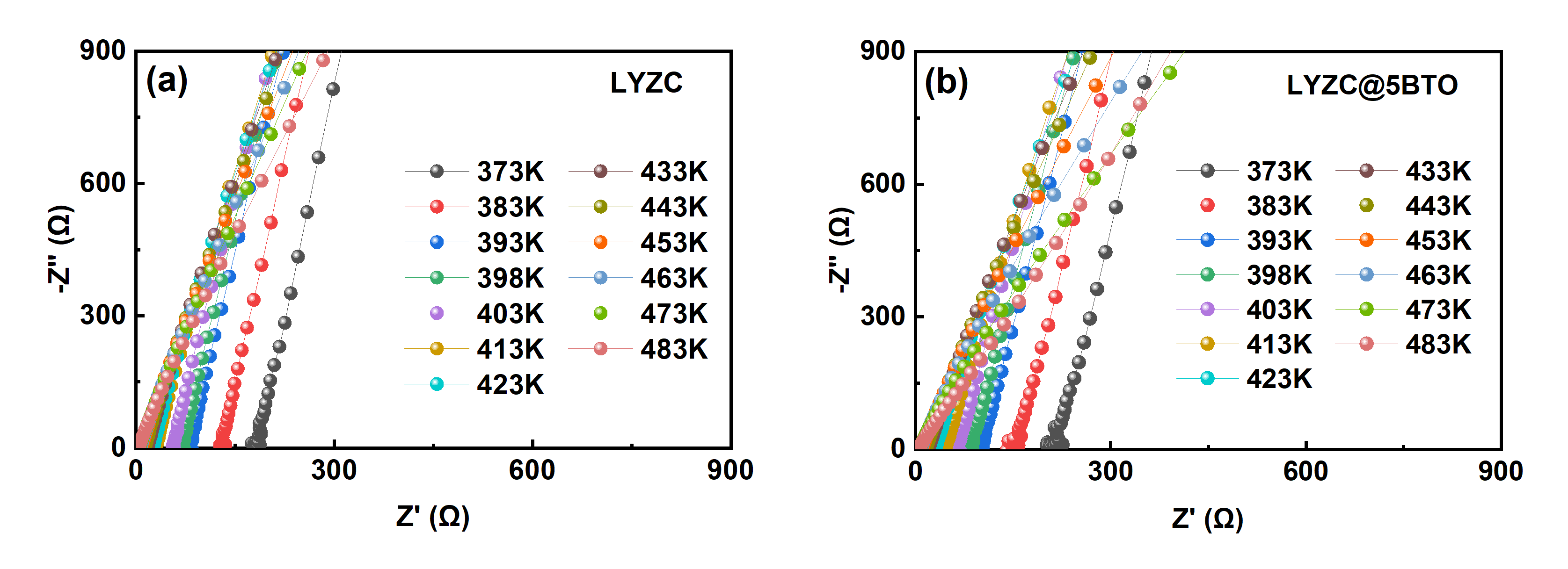


**Fig. S8** The Nyquist plots of **(a)** LYZC and **(b)** LYZC@5BTO measured over the temperature range of 0 ℃ to 110 ℃


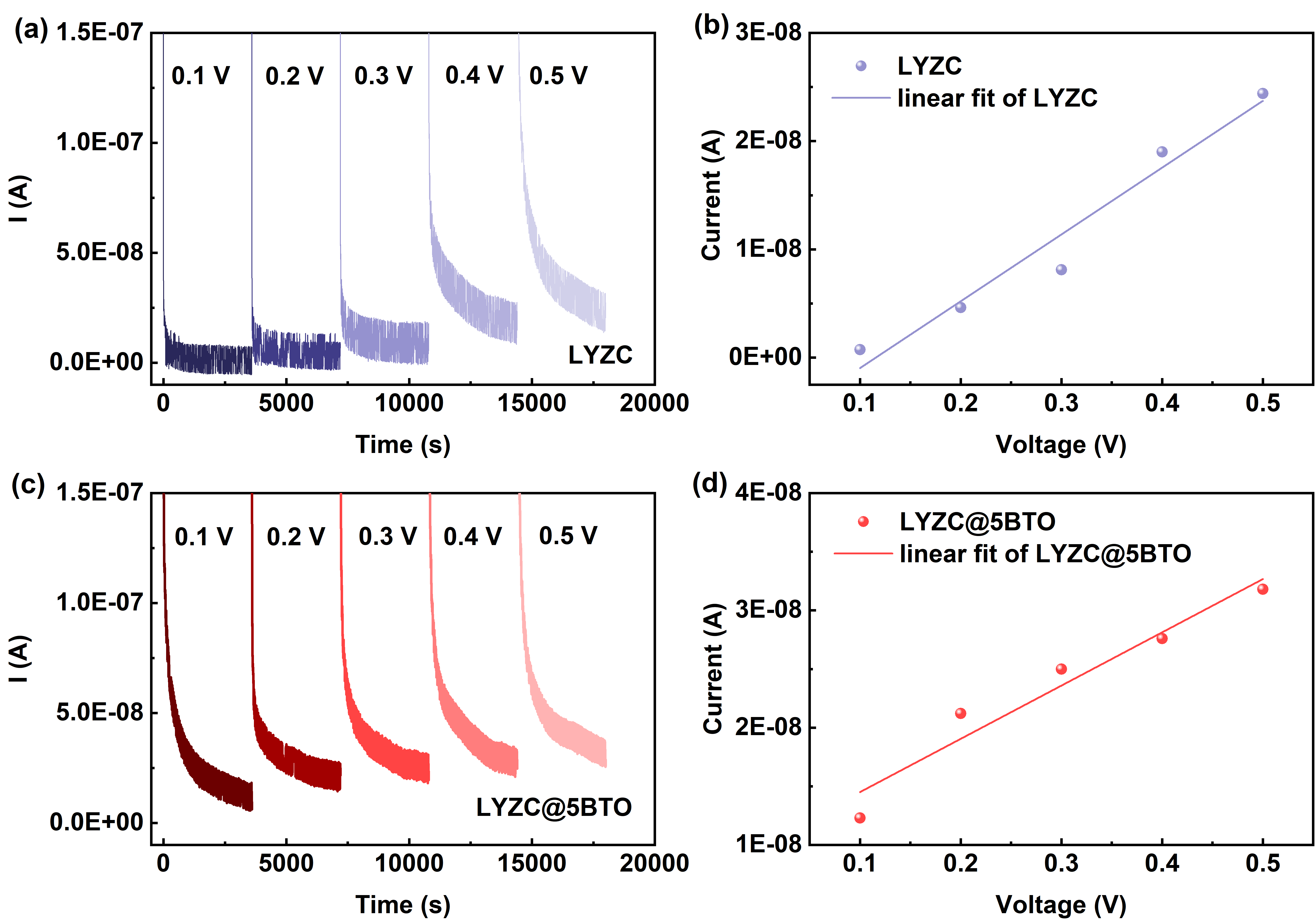


**Fig. S9** DC polarization curves of **(a)** LYZC and **(c)** LYZC@5BTO at different voltages from 0.1-0.5 V. Equilibrium current response of **(b)** LYZC and **(d)** LYZC@5BTO at different voltages. The electronic conductivity of LYZC and LYZC@5BTO determined by direct current (DC) polarization measurements from 0.1-0.5 V are 5.4 × 10^-9^ S cm^-1^ and 7.3 × 10^-9^ S cm^-1^, respectively

**
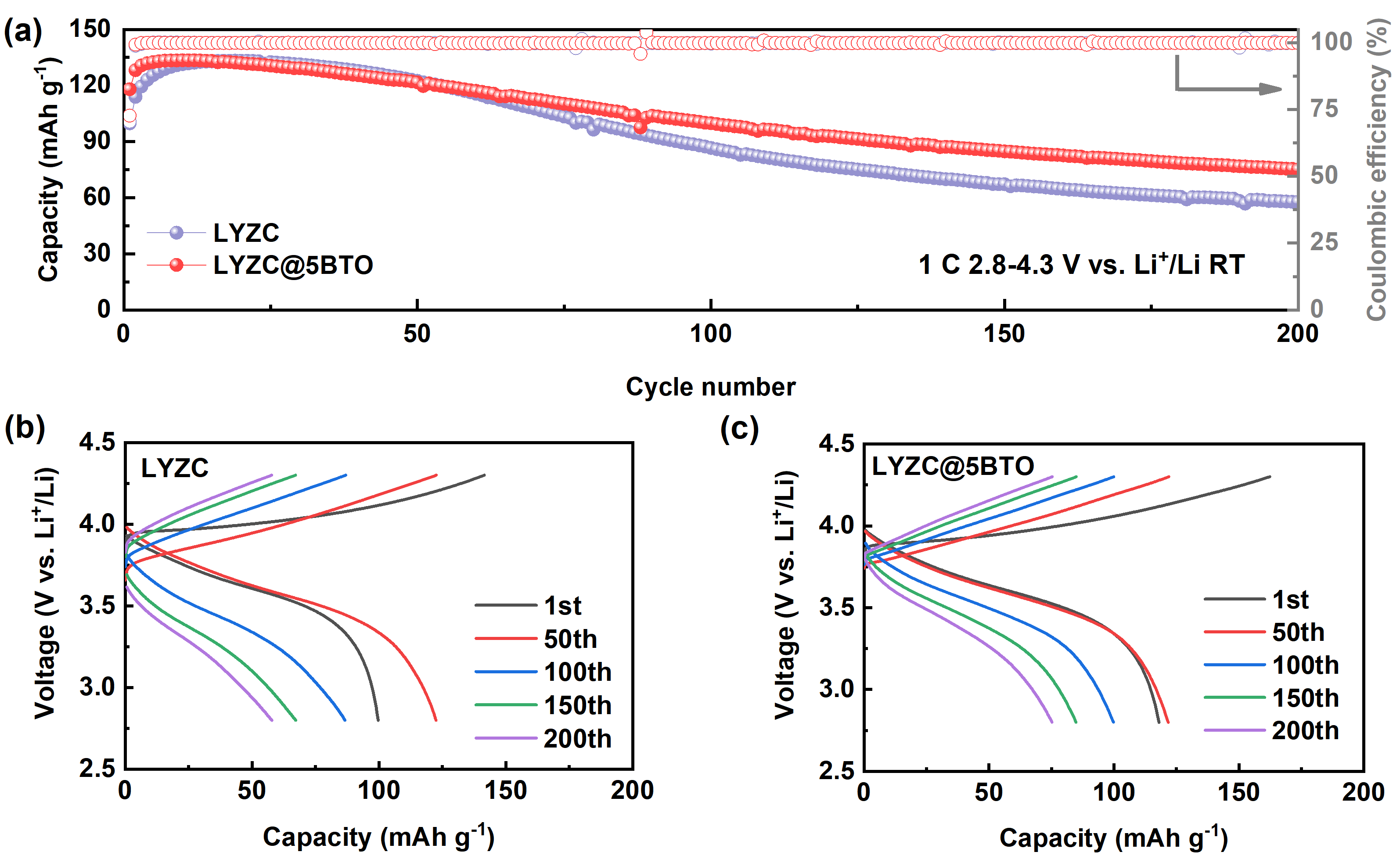
**

**Fig. S10 (a)** Cycling performance of LYZC and LYZC@5BTO at 1 C within the voltage range of 2.8-4.3 V vs. Li^+^/Li and **(b, c)** corresponding discharge profiles at different cycle numbers of LYZC and LYZC@5BTO respectively

**
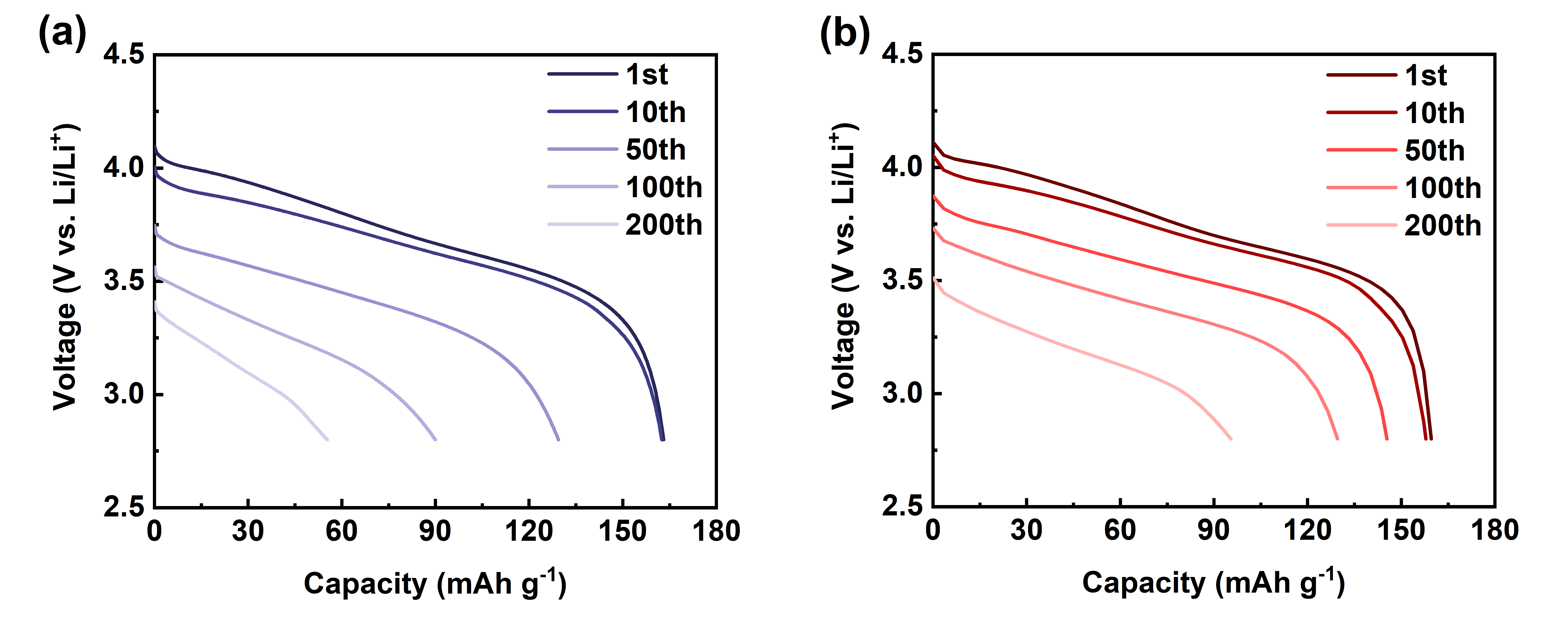
**

**Fig. S11** Discharge profiles of **(a)** LYZC and **(b)** LYZC@5BTO at 1 C at different cycle numbers


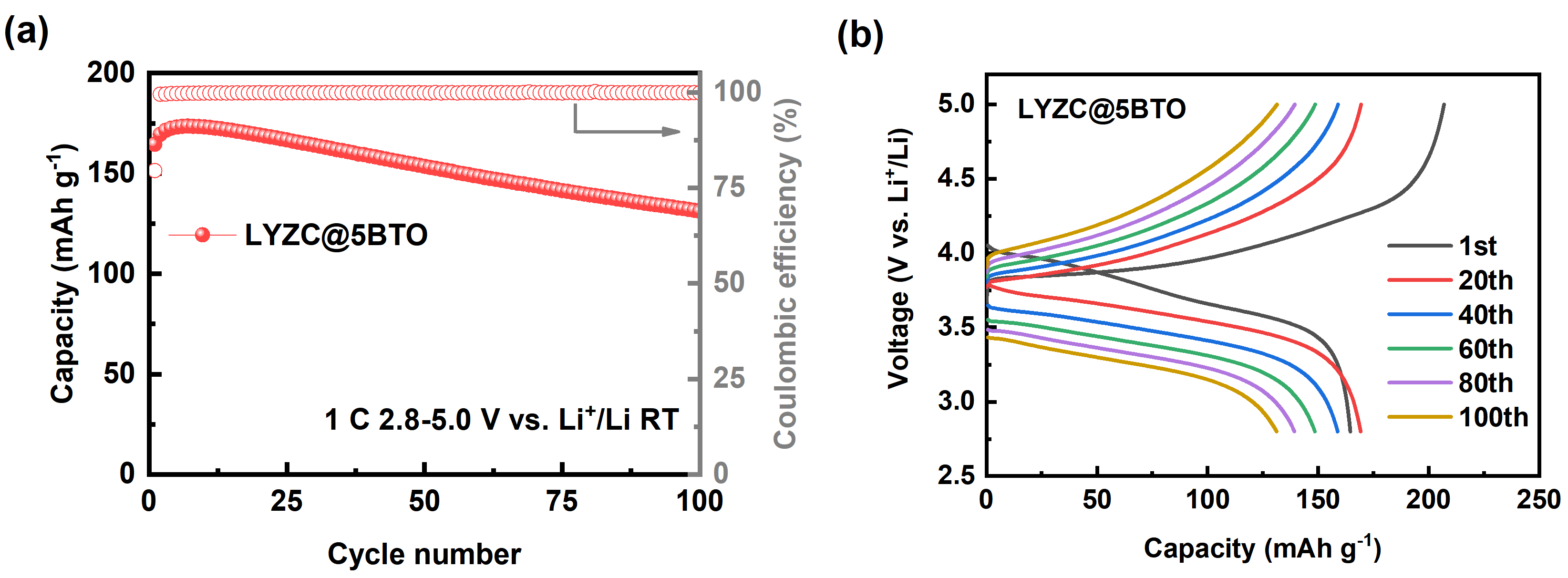


**Fig. S12 (a)** Cycling performance of LYZC@5BTO at 1 C within the voltage range of 2.8-5.0 V vs. Li^+^/Li and **(b)** corresponding discharge profiles at different cycle numbers


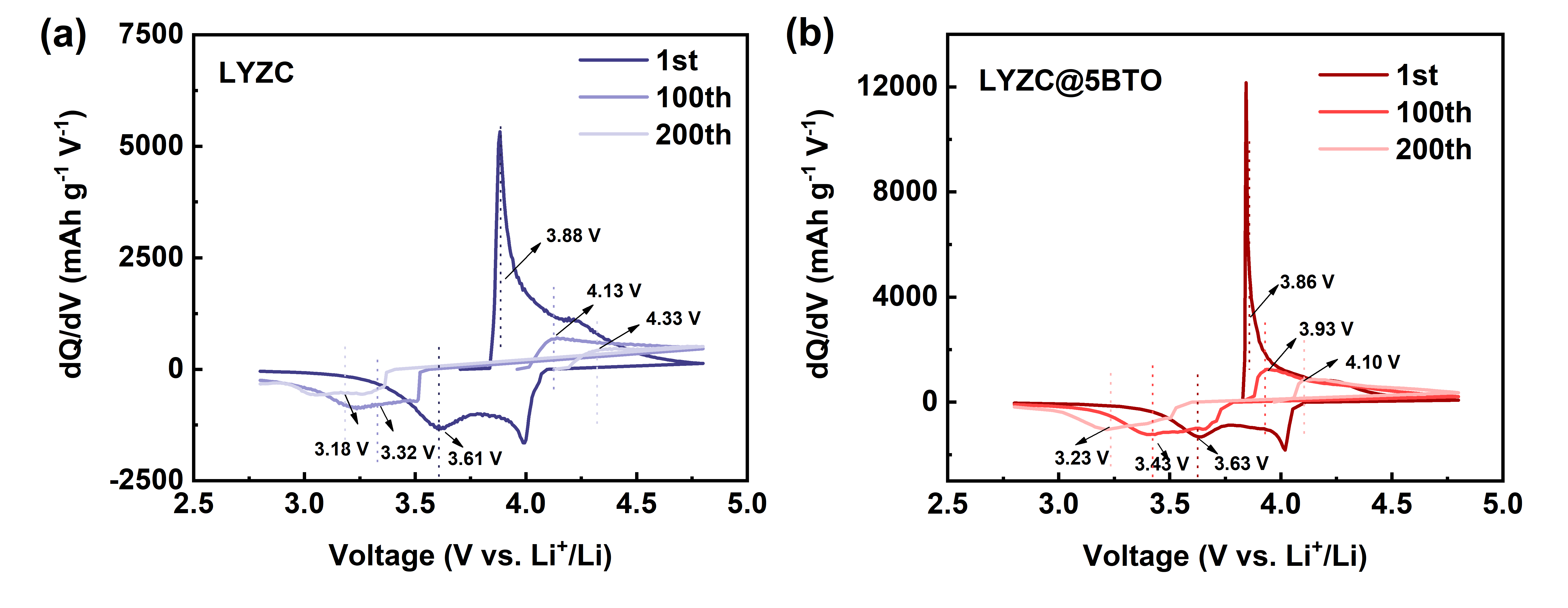


**Fig. S13** dQ/dV curves of **(a)** LYZC and **(b)** LYZC@5BTO at 1 C for the ASSBs at different cycle numbers


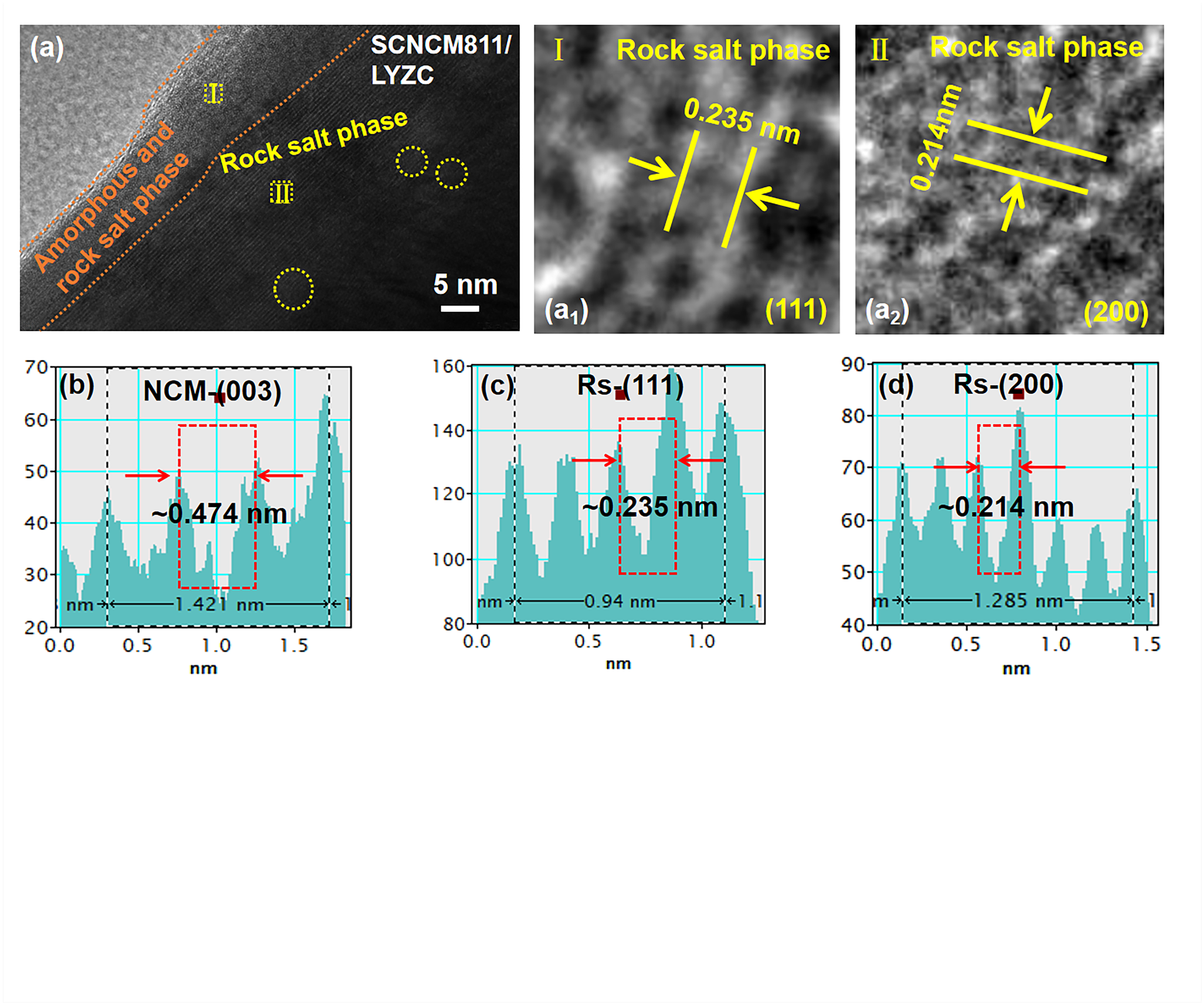


**Fig. S14** **(a)** High-resolution TEM image of SCNCM811/LYZC composite after 200 cycles with **(a_1_, a_2_)** lattice plane images of Rock-salt (Rs) phase-(111) and (200). The lattice spacing of **(b)** NCM-(003), **(c)** Rs phase-(111), and **(d)** Rs phase-(200) obtained by FFT transformation of high-resolution TEM


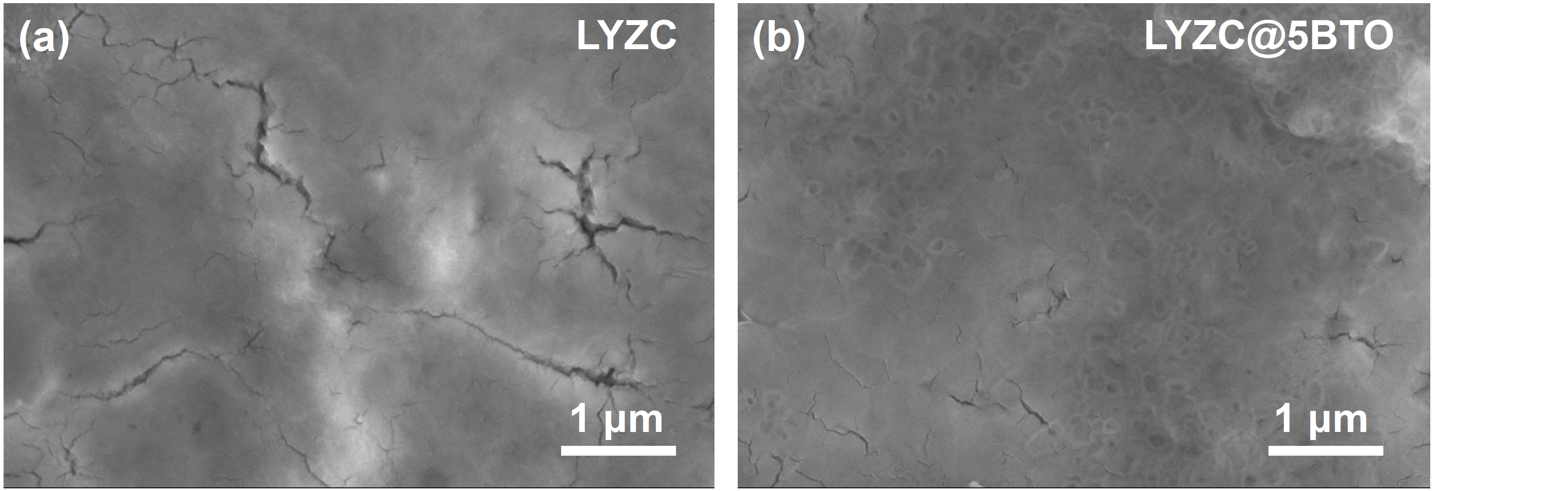


**Fig. S15** SEM images of **(a)** the SCNCM811/LYZC composite cathode and **(b)** SCNCM811/LYZC@5BTO composite cathode after 200 cycles

**Table S1** The Rietveld refinement result of LYZC@*x*BTO (*x* = 0, 2.5, 5, 7.5)

| **Samples** | **Phase 1 (wt)** | **Phase 2 (wt)** | **a (Å)** | **b (Å)** | **c (Å)** | **c/a** |
| --- | --- | --- | --- | --- | --- | --- |
| LYZC | 1.000 | 0.000 | 11.125 | 11.125 | 6.007 | 0.540 |
| LYZC@2.5BTO | 0.976 | 0.024 | 11.127 | 11.127 | 5.994 | 0.539 |
| LYZC@5BTO | 0.948 | 0.052 | 11.139 | 11.139 | 6.010 | 0.540 |
| LYZC@7.5BTO | 0.922 | 0.078 | 11.121 | 11.121 | 6.008 | 0.540 |

**Table S2** Atomic coordinates, atomic proportions, and isotropic displacement parameters of SCNCM811/LYZC composite after 200 cycles obtained from Rietveld refinement

| **Atom ncm** | **Type** | **x** | **y** | **z** | **Occ.** | **U_iso_(Å^2^)** |
| --- | --- | --- | --- | --- | --- | --- |
| Li1 | Li+1 | 0 | 0 | 0.00000 | 0.943 | 0.0021 |
| Li2 | Li+1 | 0 | 0 | 0.50000 | 0.057 | 0.0384 |
| Ni1 | Ni+3 | 0 | 0 | 0.00000 | 0.057 | 0.0164 |
| Ni2 | Ni+3 | 0 | 0 | 0.50000 | 0.743 | 0.0019 |
| Co1 | Co+3 | 0 | 0 | 0.50000 | 0.100 | 0.0030 |
| Mn1 | Mn+3 | 0 | 0 | 0.00000 | 0.100 | 0.0093 |
| O1 | O-2 | 0 | 0 | 0.23172 | 1.000 | 0.0059 |

a = b = 2.84036 Å, c=14.38932 Å

**Table S3** Atomic coordinates, atomic proportions, and isotropic displacement parameters of SCNCM811/LYZC@5BTO composite after 200 cycles obtained from Rietveld refinement

| **Atom ncm** | **Type** | **x** | **y** | **z** | **Occ.** | **U_iso_****(Å^2^)** |
| --- | --- | --- | --- | --- | --- | --- |
| Li1 | Li+1 | 0 | 0 | 0.00000 | 0.952 | 0.0530 |
| Li2 | Li+1 | 0 | 0 | 0.50000 | 0.048 | 0.0037 |
| Ni1 | Ni+3 | 0 | 0 | 0.00000 | 0.048 | 0.0041 |
| Ni2 | Ni+3 | 0 | 0 | 0.50000 | 0.752 | 0.0127 |
| Co1 | Co+3 | 0 | 0 | 0.50000 | 0.100 | 0.0364 |
| Mn1 | Mn+3 | 0 | 0 | 0.00000 | 0.100 | 0.0084 |
| O1 | O-2 | 0 | 0 | 0.23172 | 1.000 | 0.0022 |

a = b = 2.83689 Å, c=14.43864 Å
